# Exploiting the Biosynthetic Potential of Type III Polyketide Synthases

**DOI:** 10.3390/molecules21060806

**Published:** 2016-06-22

**Authors:** Yan Ping Lim, Maybelle K. Go, Wen Shan Yew

**Affiliations:** 1 Department of Biochemistry, Yong Loo Lin School of Medicine, National University of Singapore, 8 Medical Drive, Singapore 117597, Singapore; bchlimy@nus.edu.sg (Y.P.L.); bchmdkg@nus.edu.sg (M.K.G.); 2 NUS Synthetic Biology for Clinical and Technological Innovation, Centre for Life Sciences, National University of Singapore, 28 Medical Drive, Singapore 117456, Singapore

**Keywords:** chalcone synthase, stilbene synthase, polyketide, precursor-directed combinatorial biosynthesis, synthetic enzymology

## Abstract

Polyketides are structurally and functionally diverse secondary metabolites that are biosynthesized by polyketide synthases (PKSs) using acyl-CoA precursors. Recent studies in the engineering and structural characterization of PKSs have facilitated the use of target enzymes as biocatalysts to produce novel functionally optimized polyketides. These compounds may serve as potential drug leads. This review summarizes the insights gained from research on type III PKSs, from the discovery of chalcone synthase in plants to novel PKSs in bacteria and fungi. To date, at least 15 families of type III PKSs have been characterized, highlighting the utility of PKSs in the development of natural product libraries for therapeutic development.

## 1. Introduction

**Figure 1:**
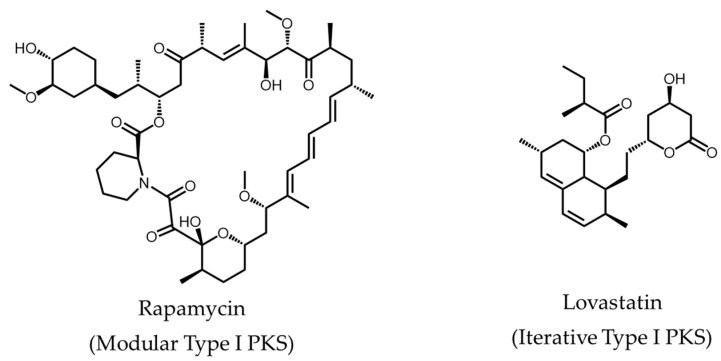

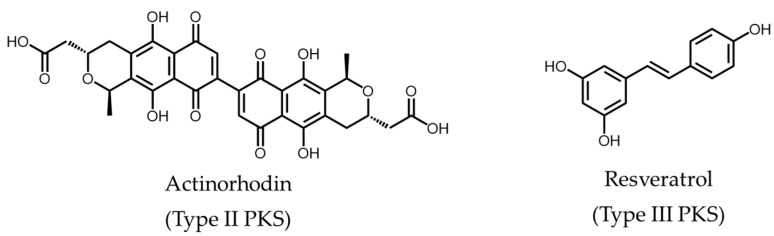
Examples of polyketides synthesized by the different classes of PKSs.

Substantial focus has been awarded to polyketide research since its initial discovery about a century ago [1]. Polyketides are structurally and functionally diverse secondary metabolites produced in bacteria, fungi, and plants. Many of these bioactive natural products have significant medical or agricultural applications [2], such as rapamycin (a macrolide immunosuppressant) [3], lovastatin (used for the treatment of hypercholesterolemia) [4], actinorhodin (an antibiotic) [5], and resveratrol (reported anti-ageing properties in model organisms) [6] (Figure 1). This highlights the vital role polyketides and their derivatives can play in the development of novel therapeutics [7]. Although chemical synthesis can be used to produce polyketide analogues [8], the chemical and structural complexity of polyketides and their derivatives prevent routine production by this route.

Insights into biosynthetic mechanisms of polyketides only achieved considerable progress from 1953 when Birch and Donovan hypothesized a potential biosynthetic pathway similar to that of fatty acids [9]. Further advancements in the polyketide field led to the consensus that polyketides are typically biosynthesized through successive decarboxylative condensations of coenzyme A (CoA)-derived units, into a complex polycyclic multi-carbon compound containing keto or hydroxyl groups [10]. Downstream modifying enzymes are then used to obtain flavonoids and other bioactive substances. Recent studies in the engineering and structural characterization of polyketide synthases (PKSs) have facilitated the use of target enzymes as biocatalysts to produce novel functionally optimized polyketides, which can serve as potential drug leads [11,12].

**Figure 2:**
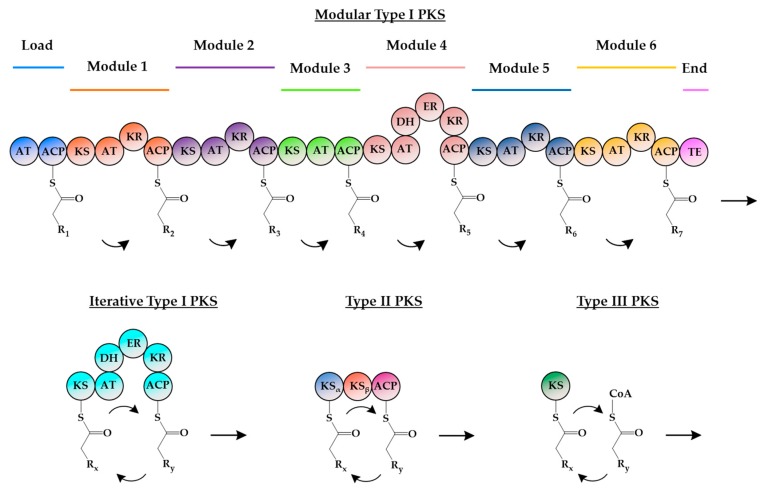
Domain organization of the different PKSs. Putative domains are represented by circles. In modular type I PKSs, functional domains are organized into several modules, with each module being responsible for a single decarboxylative condensation step in polyketide formation. For iterative type I PKSs, the functional domains are clustered in a single module, and each domain is used repeatedly during polyketide synthesis. Type II PKSs are dissociable multi-enzyme complexes, with each protein bearing a single and independent catalytic domain that is used iteratively during polyketide formation. Reactions by type III PKSs are also iterative but do not require an ACP for the attachment of the growing polyketide chain. AT: Acyltransferase; ACP: Acyl carrier protein; KS: Ketosynthase; KR: Ketoreductase; DH: Dehydratase; ER: Enoyl reductase; TE: Thioesterase; CoA: Coenzyme A.

Generally, PKSs are grouped into three classes: type I, II, or III PKS (Figure 2) [2]. Type I PKSs are large multi-domain polypeptides which can function in either a modular or iterative manner, shuttling substrates between functional domains via acyl carrier proteins (ACPs). In modular type I PKSs, functional domains are organized into several modules, with each module being responsible for a single decarboxylative condensation step in the formation of the polyketide [2,13]. Conversely for iterative type I PKSs, the functional domains are clustered in a single module; hence, each domain is used repeatedly during polyketide synthesis [14]. Type II PKSs are dissociable multi-enzyme complexes, with each protein in the complex bearing a single and independent catalytic domain that is used iteratively during polyketide formation [13]. Type III PKSs are homodimeric enzymes and are structurally simpler and mechanistically different from type I and II PKSs. The relative simplicity, versatility, and unusually broad substrate specificity of type III PKSs make them ideal candidates for the engineering of biocatalysts, and in doing so, access to bioactive polyketide compound libraries can be readily made available [15].

This review highlights recent progress in the understanding of type III PKSs (in particular with regards to the diversity of naturally occurring reactions and products discovered), and summarizes strategies for exploiting the biosynthetic potential of type III PKSs. Approaches based on random mutagenesis or site-directed mutagenesis of active site cavities to engineer the substrate specificity and activity of type III PKSs will also be discussed. These studies serve as a primer towards the development of novel and valuable polyketides with therapeutic potential.

## 2. Insights into Type III PKSs

Type III PKSs were previously only found in plants, but in the past 15 years, more bacterial type III PKSs were characterized [16,17,18]; fungal type III PKSs were only recently discovered [19,20,21]. Unlike the type I and II PKSs, type III PKSs generally utilize CoA thioesters as substrates and do not entail the involvement of ACP domains. Furthermore, type III PKSs are able to accomplish an entire series of decarboxylative condensations and cyclization reactions in a single active site [2]. By having a simple architecture, it enables type III PKSs to be amenable to *in vitro* manipulation and examination.

Although type III PKSs have been studied since the 1980s [22], structural information, on how a single active site can direct the chemistry of several decarboxylative condensations and subsequent cyclization of the polyketide, was only made known after the determination of the crystal structure of chalcone synthase (CHS) from *Medicago sativa* (commonly known as alfalfa) [23]. This was the first crystal structure of a PKS and it facilitated a framework for engineering type III PKSs to generate novel polyketides.

### 2.1. Chalcone Synthases and the Basis of Polyketide Synthesis

**Scheme 1:**
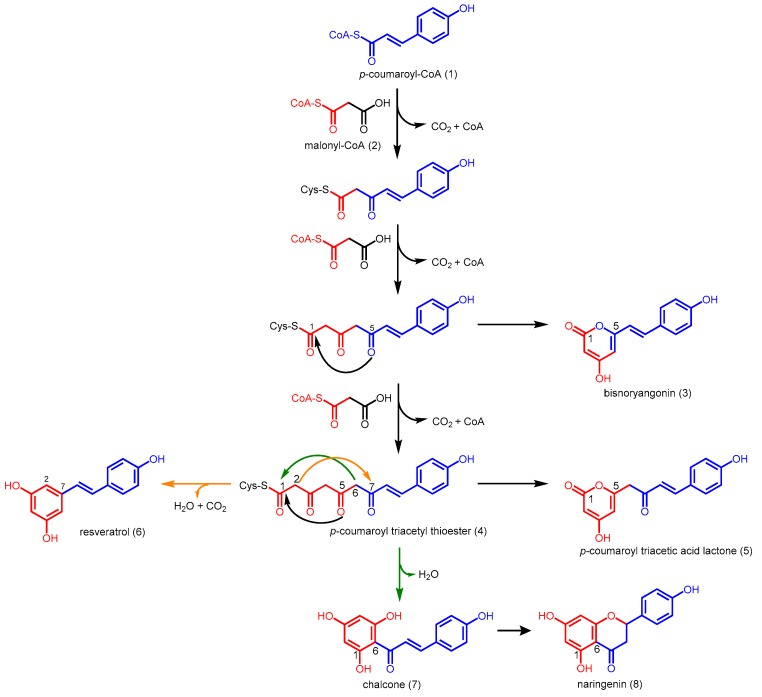
Chalcone and naringenin synthesis catalyzed by *M. sativa* CHS or *Pinus sylvestris* STS.

CHSs are the most well studied type III PKSs as they are ubiquitous in higher plants and essential for plant metabolism and defense [15]. Based on the structure of CHS from alfalfa (PDB ID: 1CGK), it was found that the symmetric dimer encapsulates a CoA-binding tunnel, a coumaroyl-binding pocket, and a cyclization pocket in order to facilitate chalcone biosynthesis [23]. Together with the conserved Cys164-His303-Asn336 catalytic triad, and the ‘gatekeeper’ Phe215 which is hypothesized to aid in the orientation of substrates during polyketide chain elongation, these connected cavities constitute the active site architecture of a typical type III PKS [24,25]. Chalcone synthesis by CHS is initiated by the loading of *p*-coumaroyl-CoA (**1**) onto the sulfhydryl group at the active site cysteine residue. The reaction then proceeds on with three iterative decarboxylative condensations of the extender substrate, malonyl-CoA (**2**), with the cysteine-bound starter unit, aided by the active site histidine and asparagine residues. After chain extension, the linear tetraketide, *p*-coumaroyl triacetyl thioester (**4**), undergoes an intramolecular C6 to C1 Claisen condensation and cyclizes into chalcone **7**, which undergoes a further Michael-type ring closure to give rise to naringenin (**8**) (Scheme 1) [24].

Numerous type III PKSs have been identified till date, and the range of reactions and product classes discovered is due to differences in intramolecular cyclizations of the linear polyketide intermediate, the number of polyketide extension steps involved, and the choice of starter and extender CoAs used [26]. The diverse polyketide scaffolds catalyzed by type III PKSs can be classified into chalcone, stilbene, pyrone, chromone, stilbenecarboxylate, bibenzyl, benzalacetone, curcuminoid, benzophenone, biphenyl, acridone, quinolone, phloroglucinol, resorcinol, and naphthalene families [24,27], respectively. By characterizing various type III PKSs, we can understand reaction mechanisms better and thus, direct the generation of unnatural polyketides using synthetic enzymology [28].

### 2.2. Stilbene Synthases and the Functional Divergence of Type III PKSs

Stilbene synthases (STSs) are found in only a small subset of unrelated plants, unlike CHSs, which are ubiquitous. Phylogenetic studies show that no ancestral STS gene was found, and STSs are likely to have evolved from CHSs independently on several occasions [29]. STSs and CHSs are structurally related and share at least 65% amino acid sequence identity [30]. These enzymes follow a similar overall reaction mechanism which involves initiation, followed by the iterative decarboxylative condensation of three units of malonyl-CoA to a starter unit, and cyclization of the linear tetraketide intermediate. Slight variations in active site residues enable STSs to catalyze an intramolecular C2 to C7 aldol condensation, followed by decarboxylation and dehydration to generate a stilbene scaffold instead of a chalcone backbone (Scheme 1) [26].

Although it has been postulated that divergence was due to some steric changes of the active site cavity affecting the orientation of the linear tetraketide intermediate, the questions of how CHS and STS can produce resveratrol (3,5,4′-trihydroxystilbene, **6**) and naringenin (**8**) respectively as minor products (Scheme 1) [30], and how decarboxylation occurs in STSs, remain unanswered. Previous homology modeling studies have shown no substantial structural or chemical differences in the STS active site, compared to CHS, and no obvious STS or CHS consensus sequence was identified [26,29]. With the report of the first STS crystal structure from *Pinus sylvestris* (Scots pine) in 2004 (PDB ID: 1U0U), the mechanism of stilbene synthesis was finally resolved [26,27].

**Figure 3:**
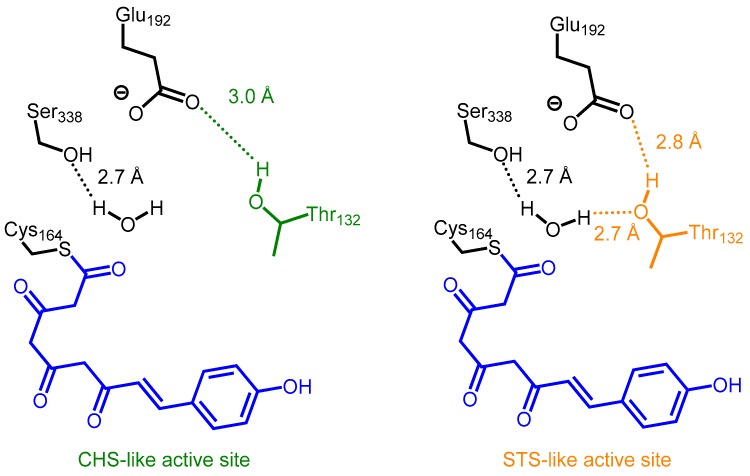
The hydrogen bond network involving Glu192, Thr132, Ser338, and H_2_O. Differences in this network switches the chemistry of the active site of *M. sativa* CHS or *P. sylvestris* STS between chalcone and stilbene synthesis.

Structure-based mutagenic transformation of alfalfa CHS into STS established that STS’s alternative cyclization specificity is due to a conformational backbone change in a short, concealed loop between residues 132 and 137 in alfalfa CHS, that spans the dimer interface in the active site. Several amino acid substitutions at buried sites adjacent to this loop bring about a slight displacement of Thr132, a residue that is conserved in both CHS and STS. Consequently, the side chain hydroxyl group of Thr132 is brought within the hydrogen bonding distance of a Ser338-stabilized water molecule adjacent to the catalytic cysteine, and a different active site hydrogen bond network involving Glu192-Thr132-H_2_O-Ser338 forms in STS (Figure 3) [24,26,31]. The nucleophilic water activated by this “aldol-switch” hydrogen bond network subsequently participates in the cleavage of the thioester bond of the cysteine-bound linear tetraketide intermediate. With the absence of the ester bond at C1, the intermediate is able to undergo a C2 to C7 aldol condensation and ultimately generates a stilbene scaffold. Intriguingly, when an ester bond is present at C1, a C6 to C1 Claisen condensation prevails [26,32]. Hence in the case of CHS and STS, the functional divergence is determined by electronic factors rather than steric effects of the active site cavity. This also explains the cross-reactivity of CHS and STS previously identified by Yamaguchi and co-workers [30]. Nevertheless, the amino acid substitutions which result in a displacement of Thr132 are varied in different STSs, probably due to gene duplication and convergent evolution. Further biochemical and structural studies on similar proteins will be required to delineate the molecular basis in greater detail [31].

### 2.3. Truncation Products and Chain Length Specificity

Some CHSs and STSs are promiscuous in terms of the number of elongation steps, and polyketide intermediates can become derailed without forming the final chalcone or stilbene product respectively [24]. These intermediates usually undergo an intramolecular C5-oxygen to C1 lactonization to give rise to heterocyclic truncation products. In the case of alfalfa CHS and Scots pine STS, derailed triketide and tetraketide intermediates form bisnoryangonin (**3**) and coumaroyl triacetic acid lactone (CTAL, **5**), respectively (Scheme 1). Although lactone derailment products can form spontaneously from linear polyketide intermediates in acidic solutions [24,30], a 2-pyrone synthase (2-PS) isolated from *Gerbera hybrida* (daisy) was identified to be able to control the final length of the polyketide and thus, was the first type III PKS to have a different product profile from CHS and STS [33,34].

**Figure 4:**
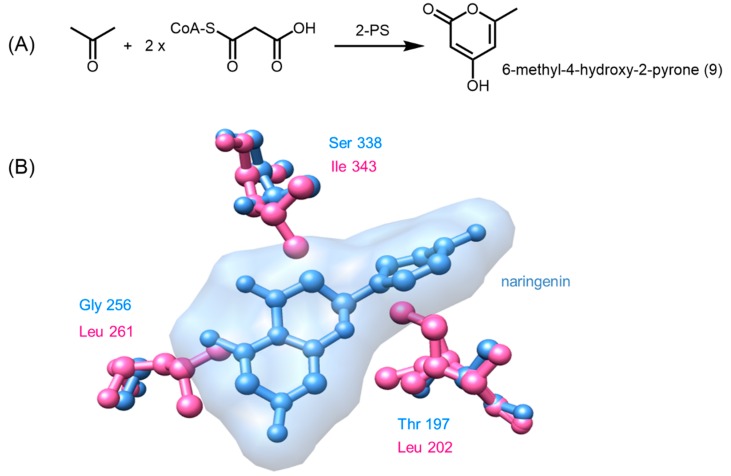
Comparing residues lining the active site of *G. hybrida* 2-PS and *M. sativa* CHS. (**A**) 2-PS catalyzes the condensation of acetyl-CoA with two units of malonyl-CoA to form 6-methyl-4-hydroxy-2-pyrone; (**B**) the structures of 2-PS (pink, PDB ID: 1QLV) and CHS (blue, PDB ID: 1CGK) were aligned to compare the active site cavity between the two structures. The presence of the three bulkier residues in 2-PS (L202, L261, and I343) show that a tetraketide such as naringenin will not fit inside the active site. Thus, only a smaller polyketide, such as 6-methyl-4-hydroxy-2-pyrone, may be formed.

Despite 2-PS and CHS sharing at least 70% amino acid sequence identity and identical catalytic residues, 2-PS catalyzes the condensation of an acetyl-CoA starter molecule with only two units of malonyl-CoA to form a triketide, which further undergoes lactonization to generate 6-methyl-4-hydroxy-2-pyrone (**9**, Figure 4) [34,35]. A closer inspection of the crystal structure of 2-PS (PDB ID: 1QLV) revealed that the active site of 2-PS is only approximately one third of the volume seen in CHS, owing to the greater steric bulk of three active site residues [24]. Intriguingly, the triple mutant T197L/G256L/S338I of alfalfa CHS was found to be functionally identical to 2-PS. The increased steric bulk at positions 197 and 338 occludes the coumaroyl-binding pocket, thus excluding big starter units such as phenylpropanoid CoA thioesters. Substitution of Gly256 in CHS with Leu261 in 2-PS reduces the size of the cyclization pocket of the active site, therefore limiting the number of chain extensions of the polyketide [24,35].

**Scheme 2:**
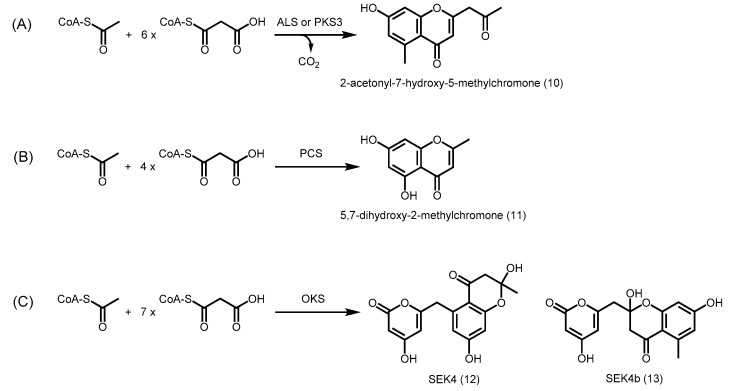
Polyketide synthesis catalyzed by *R. palmatum* ALS, *A. arborescens* PKS3, *A. arborescens* PCS, and *A. arborescens* OKS. (**A**) ALS or PKS3 catalyzes the condensation of one unit of acetyl-CoA and six units of malonyl-CoA, generating 2-acetonyl-7-hydroxy-5-methylchromone; (**B**) PCS catalyzes the condensation of one unit of acetyl-CoA and four units of malonyl-CoA, generating 5,7-dihydroxy-2-methylchromone; (**C**) OKS catalyzes the condensation of one unit of acetyl-CoA and seven units of malonyl-CoA to form SEK4 and SEK4b. Acetyl-CoA is biosynthesized from decarboxylation of malonyl-CoA.

Conversely, modifications in the active site cavity may also enable the formation of longer polyketides. Aloesone synthase (ALS) from *Rheum palmatum* (rhubarb) catalyzes six successive decarboxylative condensations of malonyl-CoA with an acetyl-CoA starter molecule, followed by an intramolecular C8 to C13 aldol condensation and decarboxylation to generate a heptaketide aloesone (2-acetonyl-7-hydroxy-5-methylchromone) (**10**) (Scheme 2). As with other plant type III PKSs, ALS shares more than 60% identity with alfalfa CHS, and maintains the Cys-His-Asn catalytic triad at positions 164, 303, and 336 respectively (numbering in alfalfa CHS) [36,37]. Interestingly, ALS lacks the conserved CHS residues at positions 197, 256, and 338, thus affecting the shape and volume of the initiation and elongation cavity of the active site. In fact, these three residues are also sterically altered in the functionally divergent *G. hybrida* 2-PS (T197L/G256L/S338I) [35]. Further research on novel type III PKSs in the medicinal plant *Aloe arborescens* (aloe) also revealed similar replacements in the pentaketide chromone synthase (PCS) (T197M/G256L/S338V) [38], heptaketide synthase (PKS3) (T197A/G256L/S338I) [39], and octaketide synthase (OKS) (T197G/G256L/S338V) [40]. These enzymes also catalyze successive decarboxylative condensations of malonyl-CoA with an acetyl-CoA starter molecule to form 5,7-dihydroxy-2-methylchromone (**11**) (pentaketide), aloesone, and SEK4 (**12**)/SEK4b (**13**) (octaketide) respectively (Scheme 2). 2-PS, PCS, PKS3, ALS, and OKS also generate their respective products by an initial decarboxylation of malonyl-CoA to produce acetyl-CoA, which is then utilized as the starter unit.

**Figure 5:**
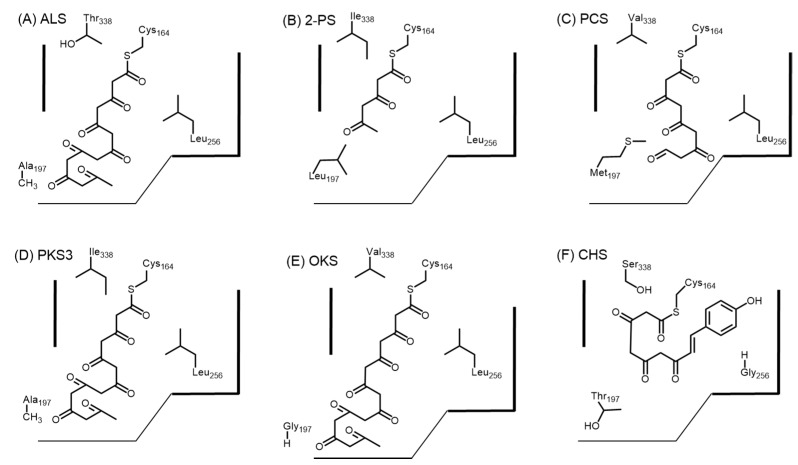
Schematic representation of the active sites of (**A**) *R. Palmatum* ALS; (**B**) *G. hybrida* 2-PS; (**C**) *A. arborescens* PCS; (**D**) *A. arborescens* PKS3; (**E**) *A. arborescens* OKS; and (**F**) *M. sativa* CHS. The residue at position 256 controls the starter unit selectivity. Bulky residues such as leucine prevents the entrance of starter acyl-CoAs such as *p-*coumaroyl CoA. The residue at position 197 controls the gate to an additional buried pocket that extends to the “floor” of the active site. Small residues such as alanine or glycine allows chain extension to form octaketides. Residue at position 338 located at the proximity of the catalytic Cys164 is at the “ceiling” of the active site cavity and this guides the growing polyketide chain to extend into the buried pocket near residue position 197. The numbering for all enzymes are based in *M. sativa* CHS.

**Figure 6:**
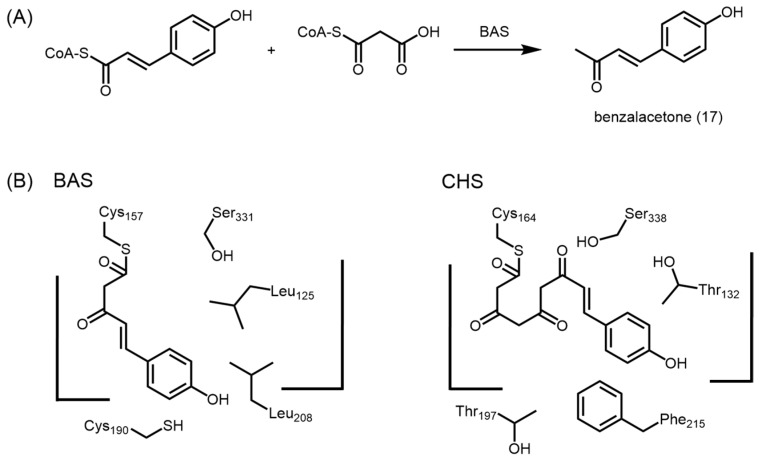
Comparison of the active sites of *M. sativa* CHS and *R. palmatum* BAS. (**A**) Synthesis of benzalacetone; (**B**) Schematic representations of the active site of BAS and CHS. The presence of Leu-208, Leu-125, and Cys-190 and the reorientation of Ser-331 in BAS changed the orientation of the starter acyl-CoA, *p-*coumaroyl-CoA, as well as the orientation of the growing polyketide intermediate, leading to the formation of benzalacetone.

Based on homology modeling with CHS, it was predicted that the active site architecture of ALS involves a ‘horizontally restricting’ bulky replacement at residue position 256 from glycine to leucine and a ‘downward expanding’ substitution at residue position 197 from threonine to alanine when compared to CHS [37]. Figure 5 shows a schematic of the effect of the residues at positions 197, 256, and 338 for *R. palmatum* ALS, *G. hybrida* 2-PS, *A. arborescens* PCS, *A. arborescens* PKS3, *A. arborescens* OKS, and *M. sativa* CHS. The residue at position 256 is found to line the initiation/elongation cavity and occupies an important site for the loading of the starter molecule, hence facilitating the use of a smaller acetyl-CoA rather than the bulky *p*-coumaroyl-CoA. The reduced steric bulk due to the threonine to alanine substitution at residue position 197 in ALS unlocks a buried pocket, thus expanding a putative cyclization cavity. Site-directed mutagenesis of ALS revealed that changing any amino acid at these positions into the CHS conserved residues (A197T, L256G, and T338S) completely prevented the formation of the heptaketide. In particular, the ALS L256G mutant was able to accept *p*-coumaroyl CoA as a starter unit to generate CTAL, thus suggesting that Gly256 controls starter unit selectivity. Further, the ALS A197T mutant was only able to form a pentaketide 2,7-dihydroxy-5-methylchromone after a C4 to C9 aldol condensation, which is a regioiosmer of the 5,7-dihydroxy-2-methylchromone (**11**) synthesized by PCS (C6 to C1 Claisen condensation) (Figure 6). Therefore, Thr197 is likely to control polyketide chain length as it is positioned at the entrance of the buried pocket. Thr338 is thought to guide the linear polyketide intermediate, which is bound to the nearby catalytic Cys164, to extend into the buried pocket, hence enabling the formation of the heptaketide.

Interestingly, further mutation of Ala197 in ALS to less bulky Gly197 in OKS (numbering in alfalfa CHS) resulted in the synthesis of SEK4 (**12**) and SEK4b (**13**, Scheme 2), the longest polyketides known to be produced by a wild-type type III PKS [37]. Mutagenesis of Met197 in PCS to Gly197 also led to the synthesis of octaketides. Moreover, the cyclization chemistry was altered from a C6 to C1 Claisen condensation of a pentaketide intermediate, to a C12 to C7 and C10 to C15 aldol condensation of an octaketide intermediate to form SEK4 and SEK4b, respectively [38]. Hence, Met197 (and Thr197 in alfalfa CHS) could be important for facilitating Claisen condensation reactions as well [24].

All in all, these findings establish that altering the size and shape of the active site cavity via site-directed mutagenesis contributes to specificity for starter molecule choice, chain elongation, and cyclization chemistry, leading to functional diversity among type III PKSs. This suggests potentially different strategies for the engineered biosynthesis of novel pharmaceutically relevant polyketides.

### 2.4. Other Members of Type III PKS from Plants

#### 2.4.1. *p-*Coumaroyl Triacetic Acid Synthase and Stilbenecarboxylate Synthase

**Scheme 3:**
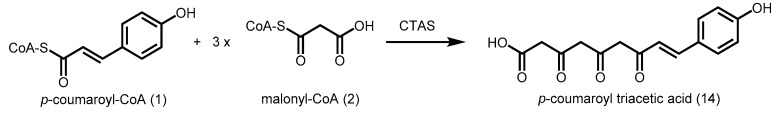
Synthesis of *p*-coumaroyl triacetic acid catalyzed by *p*-coumaroyl triacetic acid synthase (CTAS) from *Hydrangea macrophylla var. thunbergii*.

*p-*Coumaroyl triacetic acid synthase (CTAS) from *Hydrangea macrophylla var. thunbergii* was found to catalyze three consecutive decarboxylative condensations of malonyl-CoA with a *p*-coumaroyl-CoA starter without cyclization, to form *p*-coumaroyl triacetic acid (**14**) (Scheme 3) [41]. A comparison between the amino acid sequences of *H. macrophylla* CHS and CTAS showed 76% identity and only 12 amino acid residues which were significantly different in charge and size. Amongst them, replacing any of the nucleophilic residues in the CHS consensus with neutral amino acid (D/E18G) and amides (E264Q, D321N, and E324Q) in CTAS might have led to the loss of cyclization activity needed for the CHS reaction. Furthermore, the CHS-conserved Thr197, which is postulated to play a role in Claisen condensation, is replaced by an asparagine residue in CTAS [24]. All these residues are promising targets for site-directed mutagenesis and such studies may provide valuable insights into product specificity.

**Scheme 4:**
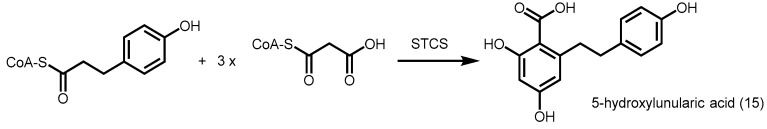
Synthesis of 5-hydroxylunularic acid catalyzed by STCS from *H. macrophylla* L.

Stilbenecarboxylate synthase (STCS) from another *Hydrangea* variety, *H. macrophylla* L., was found to utilize *p*-hydroxyphenylpropionyl-CoA as a starter and carry out three consecutive decarboxylative condensations of malonyl-CoA, followed by a C2 to C7 aldol condensation with retention of the terminal carboxyl group to form 5-hydroxylunularic acid (**15**) (Scheme 4) [42]. Although not much is known about the mechanism of action, the identification of this new class of type III PKS adds possibilities to direct the synthesis of new polyketides.

#### 2.4.2. Bibenzyl Synthase

**Scheme 5:**
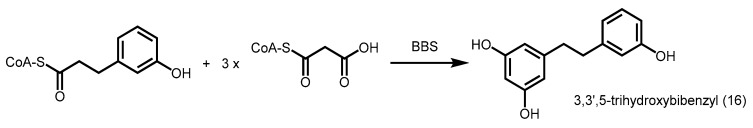
Synthesis of 3,3′,5-trihydroxybibenzyl catalyzed by BBS from a *Phalaenopsis* orchid.

Bibenzyl synthase (BBS) cloned from a *Phalaenopsis* orchid utilizes *m*-hydroxyphenylpropionyl-CoA as a starter unit and catalyzes the decarboxylative condensation of three malonyl-CoA units, followed by an aldol condensation and decarboxylation to give 3,3′,5-trihydroxybibenzyl (**16**) (Scheme 5) [43].

A comparison of amino acid sequences revealed that BBS preferred *m*-substituted derivatives of dihydrocinnamoyl-CoA to their cinnamoyl-CoA counterparts because Val98 is replaced by alanine in BBS. This substitution is also seen in other divergent type III PKSs, such as *Hordeum vulgare* (barley) homoeriodictyol/eriodictyol synthase and *Ruta graveolens* acridone synthase, which are known to accept starters containing *m*-substituted phenyl rings [24]. Furthermore, Gly211 is replaced by threonine in BBS: this small-to-large mutation is thought to alter the shape of the coumaroyl-binding pocket, as this residue is situated at the front of the active site cavity, just beneath the ‘gatekeeper’ phenylalanine positioned at the end of the CoA-binding tunnel. Derivatives of dihydrocinnamoyl-CoA possess reduced propanoid moieties and have increased conformational flexibility, giving them more advantage over their rigid counterparts in binding to a contorted coumaroyl-binding pocket in BBS. The flexibility of the tetraketide intermediate due to saturation of the propanoid moiety may also aid it to attain a folded conformation suitable for cyclization more easily within a slightly contorted active site. More structural studies are needed to provide insights into the substrate selectivity and mode of cyclization of the intermediates in BBS.

#### 2.4.3. Benzalacetone Synthase

Unlike *M. sativa* CHS, benzalacetone synthase (BAS) from *R. palmatum* is unable to generate the truncation products of the CHS reaction, bisnoryangonin and CTAL. Instead, BAS catalyzes the one-step decarboxylative condensation of malonyl-CoA to *p*-coumaroyl-CoA, followed by another decarboxylation to form benzalacetone (*p*-hydroxyphenylbut-3-ene-2-one) (**17**) (Figure 6A) [44]. The biosynthesis of benzalacetone in *R.* *palmatum* was attributed to the replacement of the ‘gatekeeper’ Phe215 with Leu208 in BAS. In alfalfa CHS, Phe215 is hypothesized to aid in orientingsubstrates during polyketide chain extension. Thus, a replacement with leucine in BAS may prevent subsequent chain extension of the diketide intermediate. In addition, the rhubarb BAS (PDB ID: 3A5R) uses an alternative coumaroyl-binding pocket to orient the starter-CoA, as the entrance to the original coumaroyl-binding pocket of the alfalfa CHS is blocked sterically in BAS by Leu125, Leu208 and Ser331 (Figure 6B) [45,46]. A single large-to-small mutation from leucine to threonine at residue position 125 of BAS back to the CHS conserved residue was found to be sufficient to unlock the buried coumaroyl-binding pocket, and the L125T BAS mutant was able to generate naringenin, CTAL and bisnoryangonin, in addition to benzalacetone [46]. Unlike STS, the crystal structure of rhubarb BAS also pointed to a hydrogen bond network between the Cys-His-Asn catalytic triad and a putative nucleophilic water molecule thought to play a role in thioester bond cleavage and the final decarboxylation step [45]. These findings provided a novel structural basis for the cleavage of the thioester bond of the enzyme-bound intermediate and the subsequent decarboxylation to form benzalacetone.

The crystal structure of PcPKS1 from *Polygonum cuspidatum* (Japanese knotweed) was also recently solved, and studies showed PcPKS1 to have both CHS and BAS activities [47]. Unlike rhubarb BAS and the classical BAS PcPKS2, the ‘gatekeeper’ Phe215 remained in PcPKS1, thus suggesting a different catalytic mechanism in the bifunctional PcPKS1. More engineering studies are needed to shed light on the functional divergence of this unique PKS.

#### 2.4.4. Type III Polyketide Synthases from *Curcuma longa*

**Scheme 6:**
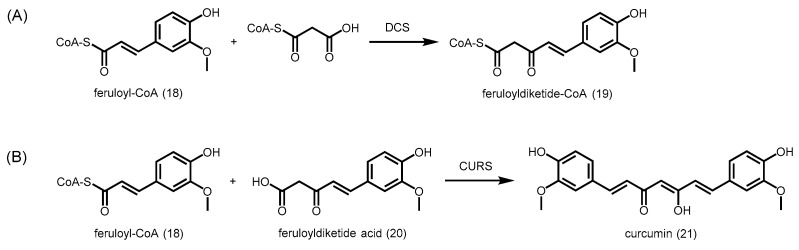
Bioynthesis of curcuminoids in *C. longa*. (**A**) DCS catalyzes the formation of feruloyldiketide-CoA, one of the rare ketides with a CoA still attached to the final product; (**B**) CURS catalyzes the formation of curcumin from feruloyl-CoA and feruloyldiketide via a rare head-to-head condensation.

The biosynthesis of curcuminoids in *C. longa* (turmeric) has been proposed to involve two type III PKSs. Diketide-CoA synthase (DCS) catalyzes the decarboxylative condensation of malonyl-CoA with feruloyl-CoA (**18**) to give rise to feruloyldiketide-CoA (**19**) (Scheme 6A). DCS is unlike most type III PKSs as it is able to release the CoA product without cleaving the thioester linkage. Subsequently, curcumin synthase (CURS) catalyzes the decarboxylative condensation of feruloyldiketide acid (**20**), the hydrolyzed β-keto acid of feruloyldiketide-CoA, with another unit of feruloyl-CoA starter to produce curcumin (**21**) (Scheme 6B) via a rarely observed head-to-head condensation of ketide units. In addition, CURS is also able to catalyze the formation of curcumin from feruloyl-CoA and malonyl-CoA, albeit at a much slower rate [48].

**Figure 7:**
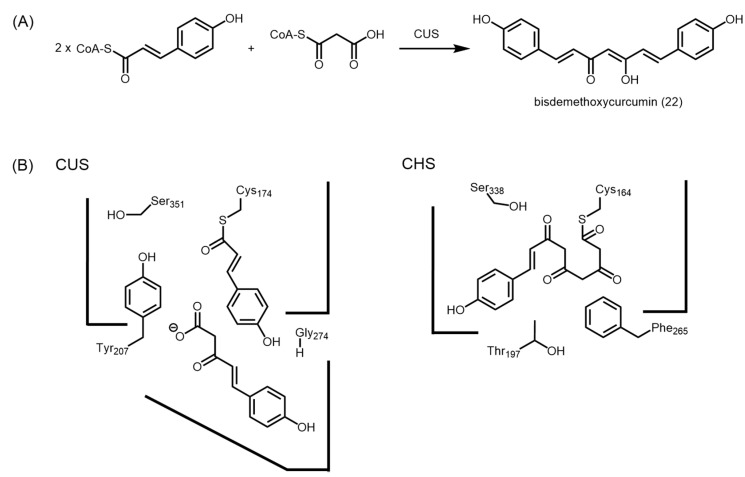
Comparison of the active sites of *M. sativa* CHS and *O. sativa* CUS. (**A**) CUS catalyzes the condensation between two units of *p*-coumaroyl-CoA and one unit of malonyl-CoA to form bisdemethoxycurcumin; (**B**) Schematic representations of the active sites of CUS and CHS. The presence of glycine at position 274 and tyrosine at position 207 orients the active site to allow longer polyketides to be generated.

Interestingly, curcuminoid synthase (CUS) from *Oryza sativa* is able to generate bisdemethoxycurcumin (**22**) from the decarboxylative condensation of two units of *p*-coumaroyl-CoA and one unit of malonyl-CoA, via a *p*-coumaroyldiketide acid intermediate (Figure 7A). Although the Cys-His-Asn catalytic triad and most conserved residues are present in the CUS active site, the crystal structure of CUS (PDB ID: 3ALE) revealed a downward expanding active site that is large enough to accommodate two starter CoAs and one extender CoA (Figure 7B) [49].

Further analysis highlighted that the CHS-conserved residues, Thr132, Thr197, Gly256, and Phe265 were replaced by Asn142, Tyr207, Met265, and Gly274, respectively in CUS. This offers no surprise as Thr132 plays a role in aldol condensation [26,31], while Thr197 is thought to direct Claisen condensation [24], and both Thr197 and Gly256 are able to direct polyketide chain lengths in other type III PKSs [35,36,37,38,39,40]. Hence by replacing these residues, the product specificity of CUS is likely to be very different from other type III PKSs. Conformational differences due to these amino acid changes resulted in a loss of the coumaroyl-binding pocket in CUS. As for Phe265, it is located near the CoA-binding tunnel in alfalfa CHS, therefore a less bulky F265G mutation in CUS is able to allow larger substrates to enter the active site [49]. In addition, the smaller glycine residue opens a gate to a downward expanding pocket, which is thought to accommodate the *p*-coumaroyldiketide acid intermediate before the second decarboxylative condensation with another unit of *p*-coumaroyl-CoA.

**Figure 8:**
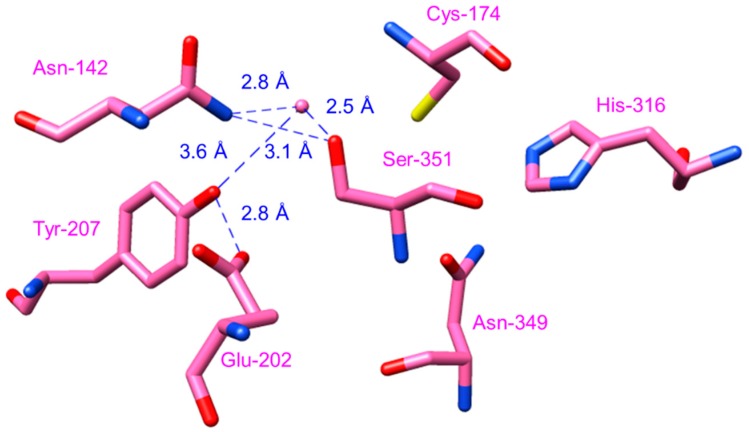
Hydrogen-bond network of CUS (PDB ID: 3ALE) consisting of Ser351, Asn142, Tyr207, Glu202, and a water molecule near the catalytic Cys174.

The crystal structure of CUS revealed a putative nucleophilic water molecule which could form a hydrogen bond network consisting of Ser351-Asn142-H_2_O-Tyr207-Glu202 near the catalytic Cys174 residue in the active site (Figure 8). This is similar to the Glu192-Thr132-H_2_O-Ser338 “aldol-switch” hydrogen bond network in STS, which participates in the cleavage of the thioester bond of the cysteine-bound linear tetraketide intermediate. The hydrogen bond network in CUS is postulated to be important for the termination of the polyketide chain extension at the diketide stage by cleaving the thioester bond of the cysteine-bound intermediate so as to produce *p*-coumaroyldiketide acid, which serves as the second extender substrate [49]. These findings provide valuable insights into the mechanism of action of CUS, the first type III PKS capable of utilizing two starters and one extender CoA to generate polyketides.

#### 2.4.5. Benzophenone Synthase and Biphenyl Synthase

**Scheme 7:**
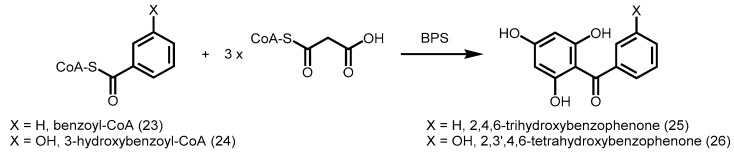
Benzophenone synthesis catalyzed by BPS from *H. androsaemum* (X = H) and *C. erythraea* (X = OH).

Among the starter-CoAs utilized by plant PKSs, benzoyl-CoA (**23**) is a rare starter unit greatly preferred only by two kinds of type III PKSs, benzophenone synthase (BPS) and biphenyl synthase (BIS) [50]. In cell cultures of *Hypericum* *androsaemum* (St. John’s wort family of plants), BPS catalyzes the decarboxylative condensation of three units of malonyl-CoA with one unit of benzoyl-CoA to form a tetraketide intermediate, which undergoes an intramolecular C6 to C1 Claisen condensation to give 2,4,6-trihydroxybenzophenone (**25**, Scheme 7) [51]. Intriguingly, *H*. *androsaemum* CHS showed 60% identity to BPS, and was able to utilize benzoyl-CoA as a starter with low activity to form benzophenone. However, BPS was unable to have any detectable activity with derivatives of cinnamoyl-CoA. Mutagenesis of three CHS residues lining the active site to their corresponding counterparts in BPS (L263M/F265Y/S338G) resulted in a mutant *H*. *androsaemum* CHS which preferred benzoyl-CoA to *p*-coumaroyl-CoA. This suggests a combination of amino acid changes can affect the coumaroyl-binding pocket of CHS, resulting in a greater substrate preference for benzoyl-CoA. Identical replacements (L263M/F265Y/S338G) were also found in the functionally similar *Garcinia mangostana* L. (mangosteen) BPS [52]. In cell cultures of the medicinal herb *Centaurium* *erythraea*, 3-hydroxybenzoyl-CoA (**24**) is the preferred starter CoA for BPS, giving rise to 2,3′,4,6-tetrahydroxybenzophenone (**26**, Scheme 7), the precursor of xanthones [53]. More structural studies are needed to provide insights as to why BPS is unable to utilize phenylpropanoid-CoA esters as starters.

**Scheme 8:**
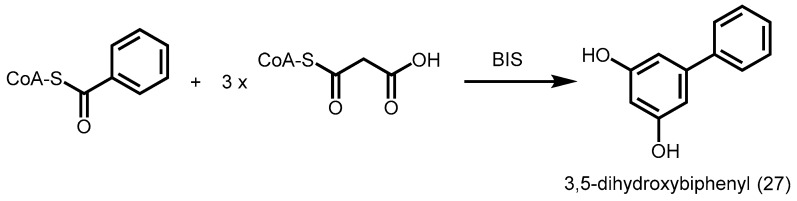
Biosynthesis of 3,5-dihydroxybiphenyl catalyzed by *S. aucuparia* BIS.

Similar to BPS, BIS from yeast extract-treated cell cultures of *Sorbus aucuparia* forms a tetraketide intermediate from one unit of benzoyl-CoA and three units of malonyl-CoA. However, the intermediate undergoes a C2 to C7 aldol condensation instead of a Claisen condensation, followed by a decarboxylation to give 3,5-dihydroxybiphenyl (**27**, Scheme 8) [54].

BIS was also not reactive towards derivatives of cinnamoyl-CoA, while *S. aucuparia* CHS was able to accept benzoyl-CoA to give the derailment product, benzoyldiacetic acid lactone. Although both STS and BIS carry out aldol condensation, Thr132 and Ser338 are replaced by alanine and glycine respectively in BIS. Hence, the “aldol-switch” hydrogen bond network consisting of Glu192-Thr132-H_2_O-Ser338 is absent in BIS. Furthermore, subtle point mutations used to disrupt this hydrogen bond network in a mutant CHS with STS activity (18xCHS), revealed that a T132A mutation led to the production of more chalcones instead of stilbenes [26]; this was surprisingly not observed in BIS and suggested a different mode of catalysis in BIS.

#### 2.4.6. Acridone Synthase and Quinolone Synthase

**Scheme 9:**
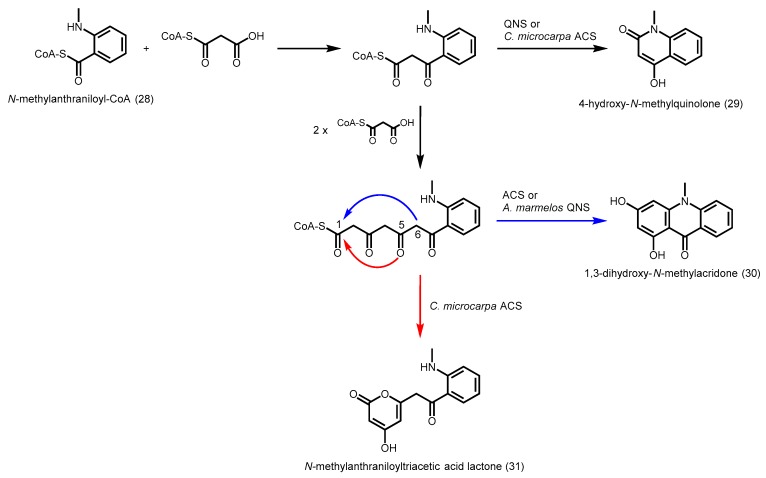
Synthesis of anthranilate-derived alkaloids catalyzed by *R. graveolens* ACS (**30** only), *A. marmelos* QNS (**29** and **30**), *C. microcarpa* QNS (**29** only), and *C. microcarpa* ACS (**29**, **30**, and **31**).

In addition to the C-C bond forming reactions, two groups of type III PKSs catalyze the C-N bond formation, resulting in the production of anthranilate-derived alkaloids. Acridone synthase (ACS) from cell cultures of *R. graveolens* catalyze the decarboxylative condensation of three units of malonyl-CoA with one unit of *N*-methylanthraniloyl-CoA (**28**), followed by a Claisen condensation to give *N*-methylaminobenzophenone, and a second ring closure to give 1, 3-dihydroxy-*N*-methylacridone (**30**, Scheme 9) [55]. Although *R. graveolens* ACS share 74% amino acid sequence identity with *R. graveolens* CHS, ACS was only able to utilize *p*-coumaroyl-CoA with low activity (10%–20%) to form naringenin. On the other hand, *R. graveolens* CHS was unable to accept the bulky *N*-methylanthraniloyl-CoA as a starter [56].

Quinolone synthase (QNS) from *Aegle marmelos* was able to catalyze the decarboxylative condensation of one unit of malonyl-CoA with one unit of *N*-methylanthraniloyl-CoA to form a diketide, which spontaneously cyclizes by amide formation to give 4-hydroxy-*N*-methylquinolone (**29**, Scheme 9). QNS was also able to form benzalacetone from *p*-coumaroyl-CoA and malonyl-CoA. Homology modeling revealed that the CoA-binding tunnel, the Cys-His-Asn catalytic triad, and most of the active site residues are maintained in *A. marmelos* QNS. However, the CHS-conserved residues Thr132, Ser133, and Phe265 are replaced by Ser132, Ala133, and Val265, respectively [57]. These substitutions are also present in *R. graveolens* ACS, hence, they are likely to play an important role in starter unit specificity [58]. The decrease in steric bulk of the residues at these three positions results in a larger active site cavity that can accommodate the bulkier *N*-methylanthraniloyl-CoA. Site-directed mutagenesis studies showed that a triple ACS mutant (S132T/A133S/V265F) was transformed into a functional CHS with some ACS side activity. Intriguingly, a similar triple QNS mutant (S132T/A133S/V265F) had no activity, but a double QNS mutant (S132T/A133S) was converted into a functional CHS that utilized *p*-coumaroyl-CoA instead of *N*-methylanthraniloyl-CoA, and the number of elongation steps was increased, leading to the production of naringenin [57]. This suggests slight differences in the active site architectures of ACS and QNS.

Recently, the crystal structures of ACS (PDB ID: 3WD7) and QNS (PDB ID: 3WD8) from *Citrus microcarpa* were solved and wide active site entrances were found in both PKSs, which enabled the access of the bulky *N*-methylanthraniloyl-CoA [59]. The V98A substitution, hypothesized to be important for *m*-substituted phenyl substrate selectivity [24], was only found in *R. graveolens* ACS [58] but not in the other ACS and QNSs identified so far [59]. *C. microcarpa* ACS was found to be a highly promiscuous enzyme with a large active site cavity, being able to accept different starter CoAs to generate chalcone, benzophenone, and phloroglucinol scaffolds in addition to the production of 4-hydroxy-*N*-methylquinolone (**29**), 1,3-dihydroxy-*N*-methylacridone (**30**), and *N*-methyl-anthraniloyltriacetic acid lactone (**31**, Scheme 9).

**Figure 9:**
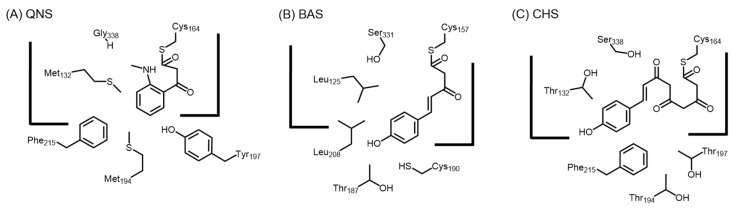
Comparison of the active sites (**A**) *C. microcarpa* QNS; (**B**) *R. palmatum* BAS; and (**C**) *M. sativa* CHS. The presence of Tyr197 and Met194 in QNS greatly reduces the active site compared to BAS and CHS; hence, only a diketide may be formed.

*C. microcarpa* QNS can only produce the diketide quinolone as the sole product, unlike *A. marmelos* QNS. Compared to *R. graveolens* ACS and *A. marmelos* QNS, similar replacements were found in *C. microcarpa* ACS (T132S/S133A/F265V), but not in *C. microcarpa* QNS (T132M/S133A/F265L). The volume of the active site cavity of *C. microcarpa* QNS (Figure 9A) is smaller compared to *R.* *palmatum* BAS (Figure 9B), due to the presence of methionine residues at positions 132 and 194, and tyrosine at position 197. These residues block the access to the coumaroyl-binding pocket [59]. Hence, *C. microcarpa* QNS is unable to utilize *p*-coumaroyl-CoA as a substrate, and chain elongation with *N*-methylanthraniloyl-CoA cannot proceed beyond the diketide stage. Intriguingly, a large-to-small tyrosine to alanine mutation at residue position 197 in *C. microcarpa* QNS mutant was able to gain the production of benzalacetone and bisnoryangonin. Site directed mutagenesis of the important active site residues Ser132, Thr194, and Thr197 in *C. microcarpa* ACS to Met, Met, and Tyr respectively resulted in a quinolone-producing enzyme which no longer produces acridone, *N*-methylanthraniloyltriacetic acid lactone, and chalcone. These results provided the first structural basis for the generation of anthranilate-derived alkaloids by type III PKSs.

#### 2.4.7. Phloroglucinol Synthase, Alkylresorcinol Synthase, and Alkylpyrone Synthase

**Scheme 10:**
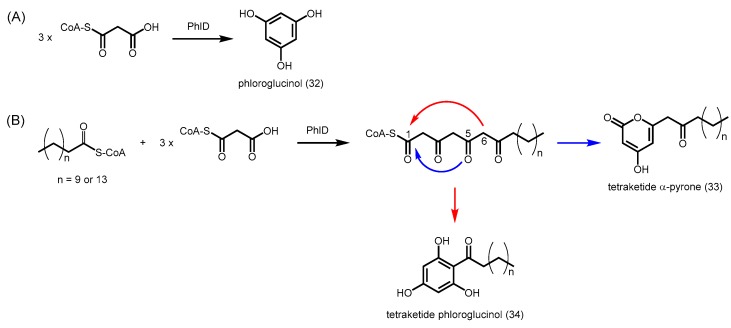
Synthesis of phloroglucinols and α-pyrones catalyzed by PhlD from *E. siliculosus*. (**A**) PhlD utilizes three units of malonyl-CoA to form the simplest phloroglucinol; (**B**) PhlD also accepts longer acyl-CoAs such as lauryl-CoA (*n* = 9) or palmitoyl-CoA (*n* = 13) to form their respective tetraketide α-pyrone and tetraketide phloroglucinol.

Most plant type III PKSs have evolved to use CoA-esters derived from phenylpropanoid metabolism as starters, but some functionally divergent PKSs are adapted to utilizing long-chain acyl-CoAs. Previous studies on *Petroselinum hortense* (parsley) CHS in the 1980s showed that type III PKSs can be promiscuous enzymes [22]. In addition to the natural substrate *p*-coumaroyl-CoA, parsley CHS was able to utilize several aliphatic CoA esters as starters, such as butyryl-CoA and hexanoyl-CoA, which were converted efficiently to acylphloroglucinols. Nevertheless, these short chain fatty acyl-CoA esters are unlikely to occur at the site of flavonoid biosynthesis, and the purpose of having activity with these substrates remained unknown. Recently, phloroglucinol synthase (PhlD) from *Ectocarpus siliculosus* (brown alga) was found to be able to synthesize phloroglucinol (**32**) from three units of malonyl-CoA (Scheme 10A), and a positive correlation was identified between reacclimation to seawater and the phloroglucinol content in cells [60]. The phloroglucinol synthase was also able to catalyze the decarboxylative condensation of three units of malonyl-CoA with one unit of lauroyl-CoA (*n* = 9) or palmitoyl-CoA (*n* = 13) to form tetraketide α-pyrones **33** and acylphloroglucinols **34** (Scheme 10B).

**Figure 10:**
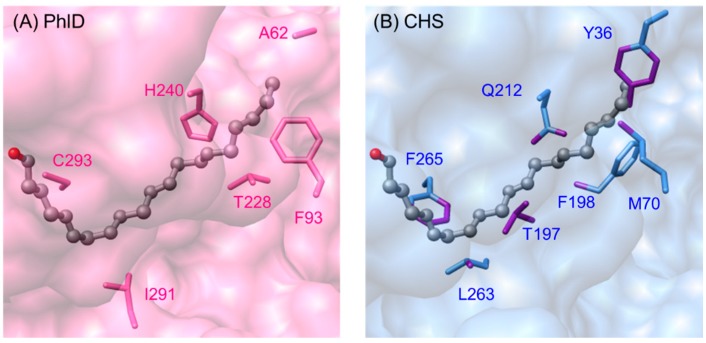
Surface view of arachidonic acid—(**A**) on the acyl-binding tunnel in *E. siliculosus* PhlD (PDB ID: 4B0N) and (**B**) superimposed on *M. sativa* CHS (PDB ID: 1CHW). Clashes between the corresponding residues in CHS that line the “tunnel” and arachidonic acid were observed (indicated by purple bonds on the residues).

PhlD was co-crystallized with arachidonic acid and the structure of the enzyme was solved at 2.85 Å resolution using molecular replacement (PDB ID: 4B0N). The structure showed that an acyl-binding tunnel is present instead of a coumaroyl-binding pocket, hence enabling the PKS to accommodate long-chain acyl-CoAs (Figure 10) [60]. The residues in the acyl-binding tunnel are not conserved in *M. sativa* CHS; when arachidonic acid is superimposed on CHS, clashes between the residues lining the site and substrate were observed (Figure 10, indicated by purple bonds of the residues).

An extra pocket is also present at the dimeric interface of the enzyme, when compared to the structures of CHS and STS. This is due to the replacement of a bulky aromatic amino acid in the cavity with Gly190 in brown alga phloroglucinol synthase, thus increasing the volume of the active site cavity. A higher resolution crystal structure is needed in order to shed more light on the molecular basis of this functionally divergent PKS.

**Scheme 11:**
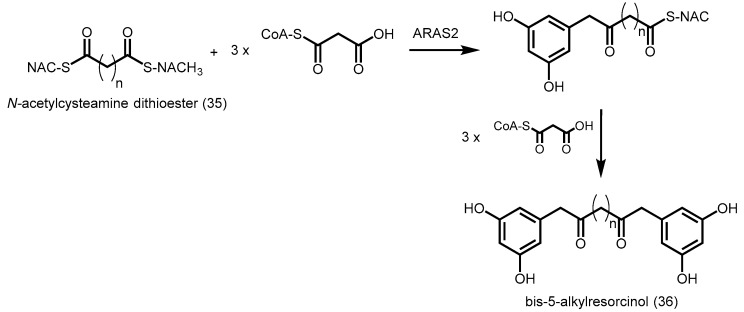
Synthesis of bis-5-alkylresorcinol catalyzed by *O. sativa* ARAS2.

Alkyl-resorcylic acid synthase 2 (ARAS2) from *O. sativa* catalyzes the synthesis of bis-5-alkylresorcinols using alkanedioic acid *N*-acetylcysteamine (NAC) dithioester (**35**) and malonyl-CoA (Scheme 11) [61]. One of the two NAC groups is first converted into a resorcinol group, generating an intermediate. ARAS2 subsequently catalyzes the condensation of malonyl-CoA with the intermediate and convert the remaining NAC group into another resorcinol group, yielding bis-5-alkylresorcinol (**36**, Scheme 11). This is analogous to the two-step reaction by curcuminoid synthase (CUS), although the mechanism of synthesis is different. ARAS2 uses two different starters and the same extenders for bis-5-alkylresorcinol biosynthesis, while CUS utilizes the same starters and two different extenders for the biosynthesis of bisdemethoxycurcumin. More structural studies are needed to provide insights into the unusual substrate specificities of ARAS2.

**Scheme 12:**
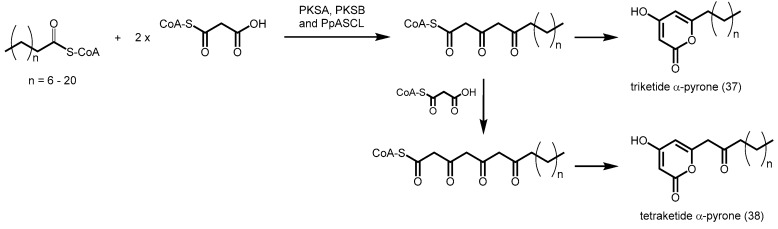
Synthesis of triketide and tetraketide α-pyrones catalyzed by *A. thaliana* PKSA and PKSB and *P. patens* PpASCL.

Recently, alkylpyrone synthases from *Arabidopsis thaliana* (PKSA and PKSB) were found to be able to produce triketide and tetraketide α-pyrones **37** and **38** from the decarboxylative condensation of malonyl-CoA with medium-chain to long-chain, and hydroxylated fatty acyl-CoAs (Scheme 12) [62]. These tetraketide α-pyrones are important for sporopollenin biosynthesis during pollen grain development, while the triketide α-pyrones are thought to be derailment products. Although these PKSs are related to CHS, they do not display CHS activity. A similar anther-specific chalcone synthase-like enzyme from *Physcomitrella patens* (PpASCL) was also discovered; the enzyme catalyzes the condensation malonyl-CoA with saturated acyl-CoAs (C6–C20), unsaturated acyl-CoAs (C16:1 or C18:1) or hydroxyl fatty acyl-CoAs to generate alkyl and hydroxyalkyl α-pyrones that are important for moss spore wall formation [63].

Both PKSA and PpASCL are also able to react with *p*-coumaroyl-CoA to yield bisnoryangonin. Based on *ab initio* multiple-threading and molecular dynamics simulation methods [63], a bulkier residue like Thr197 in alfalfa CHS is replaced by a smaller Gly205 in PKSA and Gly225 in PpASCL, hence potentially widening the entrance of the acyl-binding tunnel. In addition, the conserved Gln212 in alfalfa CHS is substituted by Ala220 in PKSA and Ala240 in PpASCL. The inability of alanine to form multiple hydrogen bonds with other active site residues may provide the structural flexibility for a larger active site cavity, thus enabling these PKSs to play a role in lipid metabolism.

### 2.5. Bacterial Type III PKSs

#### 2.5.1. 1,3,6,8-Tetrahydroxynaphthalene Synthase (THNS)

**Scheme 13:**
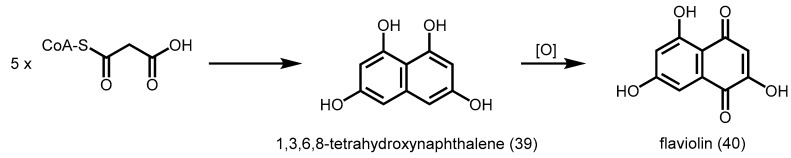
Synthesis of 1,3,6,8-tetrahydroxynaphthalene and flaviolin by *S. griseus* RppA.

Unlike plant type III PKSs, bacterial type III PKSs usually share less than 50% identity with each other and with plant type III PKSs. Bacterial type III PKSs were first characterized from the Gram-positive *Streptomyces griseus*. RppA showed 30% identity to plant CHSs, and was able to utilize malonyl-CoA as a starter and catalyze the decarboxylative condensation of another four units of malonyl-CoA to form a pentaketide, which undergoes dual cyclization reactions and decarboxylation to generate 1,3,6,8-tetrahydroxynaphthalene (THN) (**39**) [16]. THN can be further oxidized non-enzymatically to yield a reddish compound, flaviolin (**40**, Scheme 13). Cys138 serves as the catalytic residue of RppA, since a C138S RppA mutant was found to have no enzyme activity.

RppA also has alkylpyrone synthase and phloroglucinol synthase activities. It accepts aliphatic acyl-CoAs (C4–C8) as starters and catalyze decarboxylative condensations of malonyl-CoA to give α-pyrones and phloroglucinols [64]. The active site cavity of RppA is bigger than most type III PKSs; it can catalyze the formation of a hexaketide, 4-hydroxy-6-(2′,4′,6′-trioxotridecyl)-2-pyrone, using one unit of octanoyl-CoA and five units of malonyl-CoA. However unlike most plant PKSs, RppA is unable to accept aromatic acyl-CoAs such as benzoyl-CoA and phenylacetyl-CoA, indicating differences in the active site cavities of RppA and plant PKSs.

**Figure 11:**
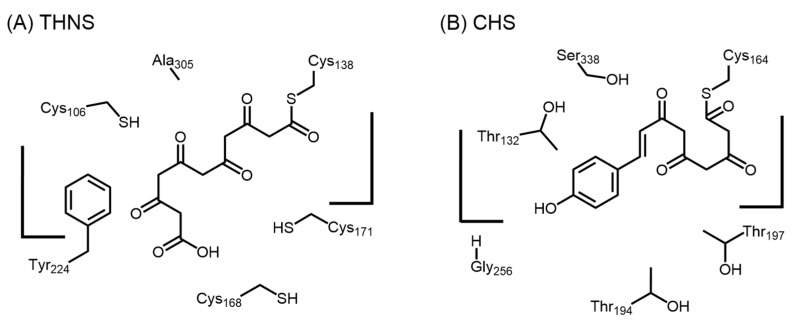
Schematic representation of the active sites of (**A**) *S. coelicolor* THNS and (**B**) *M. sativa* CHS.

A similar THNS was also found in *Streptomyces coelicolor* A3(2), although the malonyl-CoA binding for THNS was less efficient compared to that of the *S. griseus* RppA [65]. The crystal structure of *S. coelicolor* A3(2) THNS (PDB ID: 1U0M) reveals a cryptic tunnel that extends into the ‘floor’ of the traditional type III PKS active site cavity [66]. This tunnel enables the selective expansion of available active site volume by allowing the aliphatic tail of starter-derived polyketide intermediates to be sequestered. In addition, the distance between Cys138 and the cryptic tunnel precludes all but the longest reaction intermediates from accessing the novel tunnel. This provides a feasible structural explanation for the ability of RppA and THNS to catalyze an extra polyketide elongation step, instead of fewer polyketide elongation steps, when primed with a longer starter such as octanoyl-CoA *in vitro*. In addition to the cryptic tunnel, less bulky residues compared to alfalfa CHS were identified at several positions in the active site, including T132C, T194C, T197C, and S338A [66]. However, Gly256 in alfalfa CHS is replaced by Tyr224 in THNS, resembling the ‘horizontally restricting’ G256L substitution seen in 2-pyrone synthase (2-PS), pentaketide chromone synthase (PCS), aloesone synthase (ALS), and octaketide synthase (OKS), which utilize acetyl-CoA instead of *p*-coumaroyl-CoA as starters. The ‘downward expanding’ conformation of THNS active site due to the cryptic tunnel and T197C substitution, and the ‘horizontally restricting’ G256Y substitution provide some insights into the ability of THNS to utilize a small starter, and yet have the required volume for the extra polyketide elongation steps and dual cyclization reactions needed for THN generation (Figure 11). Furthermore, the replacement of Thr132 in alfalfa CHS with Cys106 in THNS leads to an additional cysteine residue in the active site, which may facilitate polyketide elongation beyond the triketide stage. We will need more liganded enzyme structures with substrate, polyketide intermediates, or product before more information regarding the substrate specificity, polyketide chain length control, and mode of dual cyclization of THNS can be ascertained.

#### 2.5.2. Phloroglucinol Synthase from *Pseudomonas fluorescens*

Phloroglucinol synthase from *Pseudomonas fluorescens* (PfPhlD) catalyzes the synthesis of phloroglucinol, a precursor of the anti-fungal 2,4-diacetylphloroglucinol (Scheme 10A). It has the same activity as *E. siliculosus* PhlD and about 40% identity with RppA [17,67]. PfPhlD was also found to display broad substrate specificity and formed α-pyrones when incubated with starters such as C4–C12 aliphatic acyl-CoAs and phenylacetyl-CoA. Similar to RppA, when primed with medium to long-chain acyl-CoAs like hexanoyl-CoA, octanoyl-CoA, and decanoyl-CoA, PhlD catalyzed extra polyketide extension steps to generate up to heptaketide products.

Homology modeling of PfPhlD using the structure of THNS as a reference highlighted the presence of a similar buried tunnel extending into the ‘floor’ of the active site cavity, which can potentially aid the binding of long chain acyl-CoAs [68]. The different reactivity towards phenylacetyl-CoA might suggest a larger and more flexible tunnel in PfPhlD that is capable of incorporating the bulky starter, compared to RppA. Saturation mutagenesis was done on important residues lining the buried tunnel, by replacing the codon encoding the residue to be mutated with the sequence ‘NNS’. This substitution with ‘NNS’ allows the replacement of the residue with all 20 amino acids, while reducing the number of premature stop codons to one (TAG) out of three. After deconvolution, three mutations M21I, H24V, and L59M, were found to significantly decrease the reactivity of PfPhlD with lauroyl-CoA, but not its physiological activity to synthesize phloroglucinol. This showed that even subtle decreases in the tunnel volume could impair the ability of PhlD to accept long chain acyl-CoAs. This highlighted the utility of a combination of rational design and random mutagenesis in changing substrate specificities, suggesting novel strategies for the combinatorial biosynthesis of unnatural polyketide libraries.

#### 2.5.3. 3,5-Dihydroxyphenylacetic Acid Synthase

**Scheme 14:**
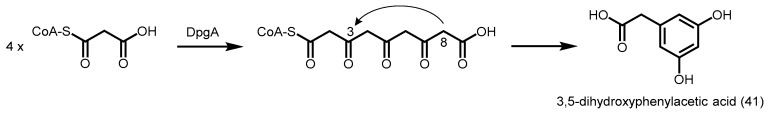
Synthesis of 3,5-dihydroxyphenylacetic acid catalyzed by *A. mediterranei* DpgA.

3,5-Dihydroxyphenylacetic acid synthase from *Amycolatopsis mediterranei* (DpgA) catalyzes the decarboxylative condensation of four units of malonyl-CoA, followed by a likely C8 to C3 aldol condensation to yield 3,5-dihydroxyphenylacetic acid (**41**, Scheme 14). The compound is a precursor of (*S*)-3,5-dihydroxyphenylglycine, an amino acid utilized in the biosynthesis of balhimycin, a vancomycin derivative [18].

Intriguingly, site-directed mutagenesis of the conserved catalytic Cys160 to alanine or serine in DpgA did not completely ablate the ability to form products [69]. When malonyl-diketidyl-CoA was used to probe how polyketide elongation and cyclization of the polyketide intermediate occurs, DpgA was found to accept malonyl-diketidyl-CoA, both as a starter and an extender, thus complicating analyses [70]. More structural studies are needed to understand how polyketide extension occurs without the catalytic cysteine, and how the C8 to C3 aldol condensation, which is unique to DpgA, is catalyzed.

#### 2.5.4. Biosynthesis of Alkylpyrones by PKS11 and PKS18

**Figure 12:**
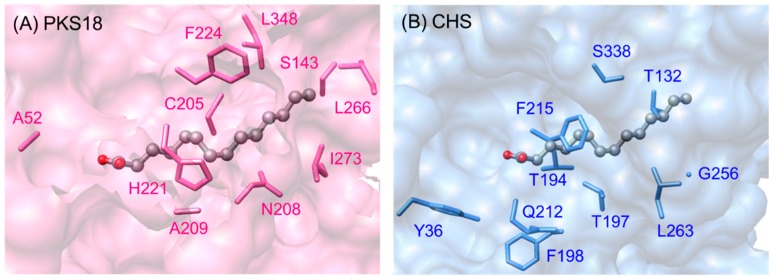
(**A**) Acyl-binding tunnel in *M. tuberculosis* PKS18 (PDB ID: 1TED); (**B**) Myristic acid (from PDB ID: 1TED) superimposed on *M. sativa* CHS (PDB ID: 1CGK). The residues on the acyl binding tunnel of PKS18 are relatively smaller (A52, S143, C205, A209) and/or more hydrophobic (N208, H221, L266, L348).

Similar to *A. thaliana* PKSA and *P. patens* PpASCL (anther-specific chalcone synthase-like enzyme), PKS11 and PKS18 from *Mycobacterium tuberculosis* were found to be able to accept medium to long-chain acyl-CoAs (C6–C20) to produce tetraketide and/or triketide α-pyrones (Scheme 12) [71]. The crystal structure of PKS18 (PDB ID: 1TED)was independently determined at the same time as the THNS structure, and the orientations of the catalytic triad (Cys175, His313, Asn346) and other functional residues were found to be similar to that of *M. sativa* CHS. Subtle differences between the primary sequences of PKS18 and *M. sativa* CHS result in the protrusion of Cys205 in PKS18 (corresponding to Thr194 in alfalfa CHS) into the putative coumaroyl-binding pocket [72]. In contrast, PKS18 was found to have a novel acyl-binding tunnel, which can bind aliphatic acyl-CoAs as starter units (Figure 12). Comparison with alfalfa CHS crystal structure showed that the entrance of the binding tunnel in CHS was blocked by Thr197, whereas the corresponding Asn208 residue in PKS18 was not extending into the tunnel. This is analogous to PKSA and PpASCL, whereby a replacement of bulky Thr197 in alfalfa CHS with a smaller Gly205 in PKSA and Gly225 in PpASCL is believed to widen the entrance of the acyl-binding tunnel, enabling them to utilize larger acyl-CoAs as starters [63].

The crystal structure of PKS11 was recently solved (PDB ID: 4JAP), and a buried hydrophobic tunnel was also revealed, together with a CoA-binding tunnel [73]. Co-crystallizations and *in vitro* enzyme assays revealed that PKS11 can catalyze the decarboxylative condensation of methylmalonyl-CoA with palmitoyl-CoA (C16) or stearoyl-CoA (C18), followed by another decarboxylative condensation of malonyl-CoA and lactonization to generate methylated alkylpyrones. The ability of PKS11 to select different extenders at different stages of polyketide elongation is intriguing, and more structural studies in this aspect will be useful for the generation of clinically important unnatural polyketides.

#### 2.5.5. Pyrone Synthases from *S. coelicolor*

**Scheme 15:**
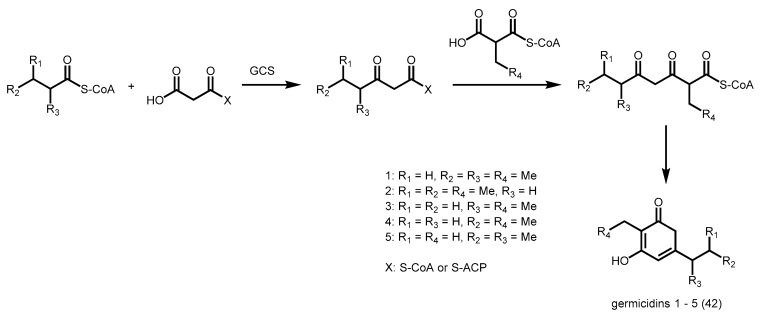
Synthesis of various germicidins catalyzed by *S. coelicolor* GCS.

Two pyrone synthases from *S. coelicolor* were recently characterized. Germicidin synthase (GCS) utilizes short to medium-chain acyl-CoAs (C4–C8) and acetoacetyl-CoA as starter units and malonyl-CoA, methylmalonyl-CoA and ethylmalonyl-CoA as extender units to generate various germicidins (**42**, Scheme 15) [74].

A type III polyketide synthase in *S. coelicolor* (locus tag: SCO7671) efficiently utilizes benzoyl-CoA and the branched *iso*-butyryl-CoA as starters to form pyrones. In addition, SCO7671 and GCS condensed malonyl-CoA with hexanoyl-ACP to yield pyrones; this is the first time type III PKSs were found to utilize acyl-ACPs [74,75]. Both PKSs are capable of accommodating huge macromolecular substrates as acyl carriers, presenting a novel means to manipulate PKSs to expand their repertoire of biosynthesized products. The crystal structure of GCS was recently solved, but more structural studies are needed before the molecular basis of the interaction between GCS and ACP can be understood [76].

#### 2.5.6. Other Bacterial Type III PKSs

**Scheme 16:**
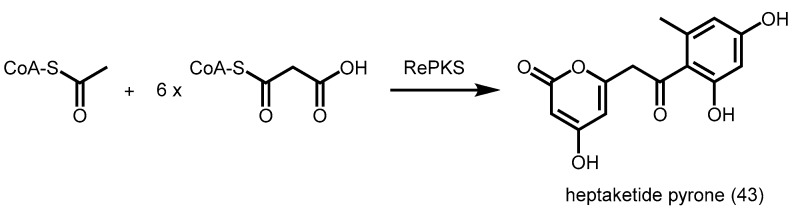
Synthesis of heptaketide pyrone from *R. etli* RePKS.

Recently, a heptaketide pyrone synthase from the soil bacterium *Rhizobium etli* (RePKS) was characterized. Unlike the *R. palmatum* aloesone synthase (ALS), RePKS undergoes dual cyclization reactions and generates a heptaketide pyrone **43** from one unit of acetyl-CoA and six units of malonyl-CoA (Scheme 16) [77]. RePKS also showed broad substrate specificities, generating triketide and/or tetraketide pyrones from acyl-CoAs (C4–C18) and benzoyl-CoA. Homology modeling highlighted that the active site cavity of RePKS is slightly larger than the active site cavity of THNS, hence partially explaining why RePKS can carry out more rounds of polyketide extension. More studies will be needed in order to provide insights into the differences in the mechanisms of action between RePKS, THNS, and *R. palmatum* ALS.

**Scheme 17:**
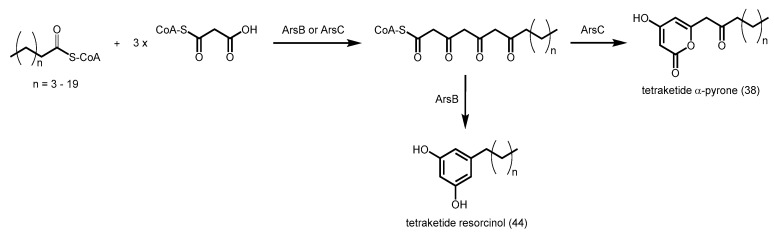
Synthesis of tetraketide α-pyrones and tetraketide resorcinols catalyzed by *A. vinelandii* ArsC and ArsB respectively.

In *Azotobacter vinelandii*, two alkylresorcinol synthases are encoded in the *ars* operon–ArsB and ArsC. They share 71% identity with each other but are enzymatically distinct PKSs. Both enzymes can utilize the same long-chain acyl-CoA as a starter and catalyze three successive decarboxylative condensations of malonyl-CoA to yield a tetraketide intermediate. Subtle differences in the active site residues of ArsB result in an intramolecular C2 to C7 aldol condensation of the intermediate, followed by a decarboxylation to produce alkylresorcinols **44** (Scheme 17), which are vital components of the cyst coat that is generated during bacterial dormancy. However for ArsC, the intermediate undergoes a C5-oxygen to C1 lactonization to form alkylpyrones (Scheme 17) [78]. ArsB was found to be able to accept a variety of acyl-CoA starters (C10–C22) while ArsC showed a wider substrate range (C6 to C22).

**Figure 13:**
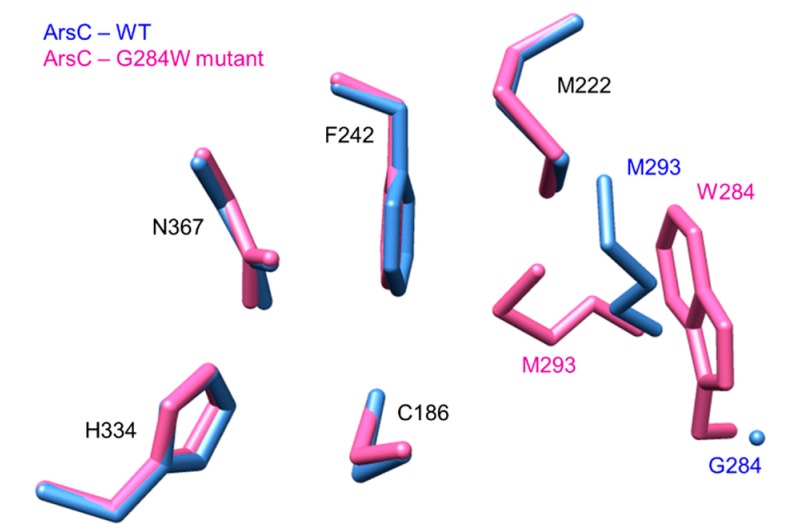
Active site cavity of ArsC-WT (blue, PDB ID: 3VS8) and ArsC-G284W mutant (pink, PDB ID: 3VS9). The Cys-His-Asn catalytic triad remained in their positions; the absence of tryptophan in position 284 allowed Met293 to swing out of the cavity, giving the growing polyketide more space.

In addition to straight chain acyl-CoAs, fatty acids bound to the ACP domain of the fatty acid synthase, ArsA, were found to be able to be directly transferred to the catalytic cysteines of ArsB and ArsC, indicating that unlike most type III PKSs, these PKSs can also utilize acyl-ACP as starters [79]. The crystal structure of ArsC (PDB ID: 3VS8) showed a hydrophobic acyl-binding tunnel similar to PKS18, explaining the ability of ArsC to utilize long-chain acyl-CoAs. Interestingly, mutating the Trp281 residue in ArsB to the corresponding Gly residue in ArsC resulted in an ArsB W281G mutant that was unable to produce any alkylresorcinol, but gained the synthesis of alkylpyrones. In contrast, the corresponding G284W mutation in ArsC resulted in a decrease in alkylpyrone production and a concomitant increase in alkylresorcinol generation [80]. This highlighted the fact that Trp281 in ArsB and Gly284 in ArsC play an important role in determining the cyclization specificity of the PKS. The crystal structure of the ArsC G284W mutant showed a significant decrease in the active site cavity volume when compared to that of the wild-type ArsC. Hence, the presence of an aromatic residue in the active sites of ArsB and the ArsC G284W mutant is likely to provide a steric wall for the tetraketide intermediates to be folded into a suitable configuration for aldol condensation to occur. The presence of the tryptophan residue at position 284 resulted in a modulation of the position of Met293 into the active site, greatly decreasing the space for polyketide synthesis (Figure 13).

**Scheme 18:**
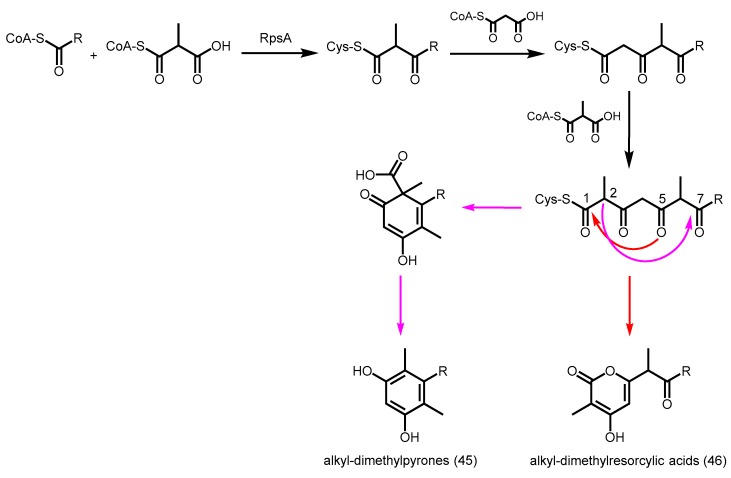
Synthesis of alkyl-dimethylpyrones and alkyl-dimethylresorcylic acids by *R. centenum* RpsA.

A type III PKS from *Rhodospirillum centenum*, *Rhodospirillum* polyketide synthase (RpsA), was characterized and found to utilize a variety of acyl-CoAs (C6–C20) as starters, followed by the decarboxylative condensations of one methylmalonyl-CoA, one malonyl-CoA, and another methylmalonyl-CoA to yield alkyldimethylpyrones **45** and/or alkyldimethylresorcylic acids **46** (Scheme 18) [81]. RpsA is the first bacterial type III PKS capable of accepting two units of methylmalonyl-CoA as extenders efficiently. More studies are needed to provide insights into the ability of RpsA to selectively control the condensation order of extenders at different stages of polyketide elongation.

### 2.6. Fungal Type III PKSs

Although most fungal polyketides such as lovastatin are synthesized by the iterative type I PKSs, fungal type III PKS genes were identified recently in the past decade. Using the alfalfa CHS amino acid sequence as a reference, four putative type III PKSs, namely chalcone synthase-like A, chalcone synthase-like B, chalcone synthase-like C, and chalcone synthase-like D (CsyA, CsyB, CsyC, and CsyD), were found in the filamentous fungus, *Aspergillus oryzae*. Similar CHS-like genes were also identified in *Fusarium graminearum* and *Neurospora crassa* (one homolog each), *Magnaporthe grisea* and *Podospora anserina* (two homologs each), *Phanerochaete chrysosporium* (three homologs each) [19], and several species of lichen fungi [82].

**Scheme 19:**
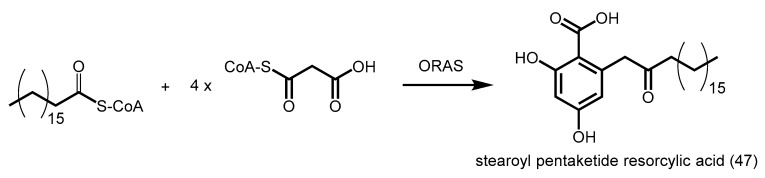
Synthesis of stearoyl pentaketide resorcylic acid catalyzed by *N. crassa* ORAS.

The first fungal type III PKS was characterized from the filamentous fungus, *N. crassa*, and showed approximately 26% identity to other plant and bacterial PKSs [83]. 2′-oxoalkylresorcylic acid synthase (ORAS) from *N. crassa* was found to catalyze the decarboxylative condensation of four units of malonyl-CoA with a unit of long-chain acyl-CoA (C18) as the preferred starter, followed by a C2 to C7 aldol condensation to generate stearoyl pentaketide resorcylic acid (**47**) (Scheme 19) [20].

**Figure 14:**
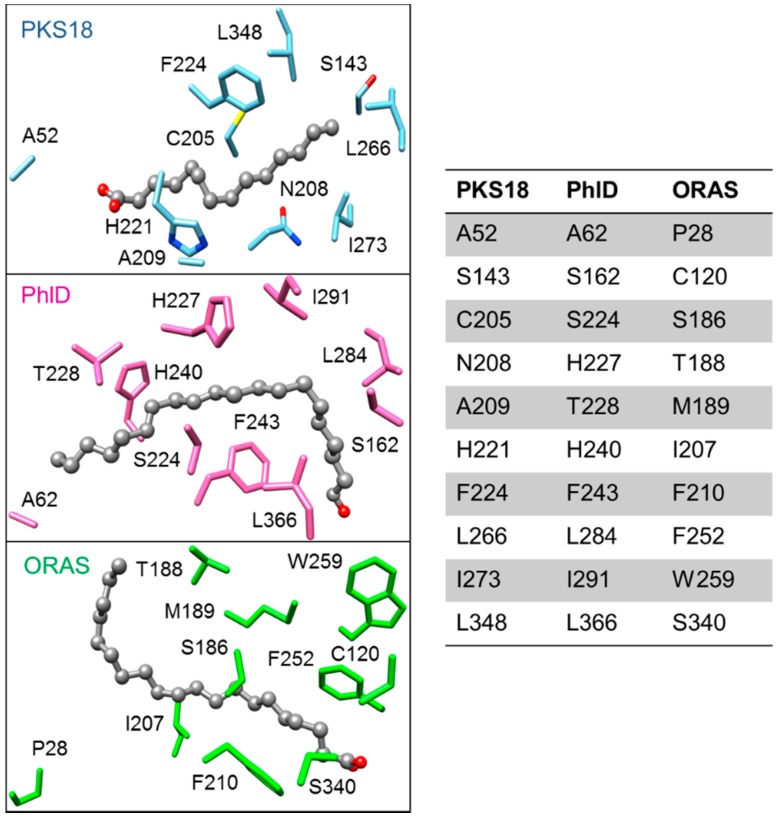
Acyl-binding tunnel of the following type III PKSs: *M. tuberculosis* PKS18 (blue, PDB ID: 1TED), *E. siliculosus* PhlD (pink, PDB ID: 4B0N), and *N. crassa* ORAS (green, PDB ID: 3EUT). The table shows the residues lining the acyl-binding tunnel of the respective PKSs. PKS18 and PhlD are more similar compared to ORAS; it is possible that these changes lining the acyl-binding tunnel lead to a different product chemistry in ORAS compared to PKS18 and PhlD.

ORAS was also capable of accepting a range of acyl-CoAs (C2–C24) as starters to produce triketide alkylpyrones (from C2 to C20 acyl-CoA substrates), tetraketide alkylpyrones (from C8–C16 acyl-CoA substrates), tetraketide alkylresorcinols (from C10–C24 acyl-CoA substrates), tetraketide alkylresorcylic acids (from C20 acyl-CoA substrate), pentaketide alkylresorcinols (from C16–C20 acyl-CoA substrates), and pentaketide alkylresorcylic acids (from C18–C20 acyl-CoA substrates). Similar to most bacterial type III PKSs, ORAS contains a second active site thiol (Cys120) at a position corresponding to the THNS Cys106, which may help in steering the polyketide reactivity [84]. The crystal structure of ORAS (PDB ID: 3EUT) also bears structural resemblance to PKS18 and *E. siluculosus* phloroglucinol synthase (PhlD), and has a deep hydrophobic acyl-binding tunnel for accommodating long-chain acyl-CoAs, along with the conserved catalytic triad (Cys152, His305, and Asn338) and the CoA-binding tunnel (Figure 14) [83]. However, several critical changes in the active site residues result in a different product chemistry for ORAS, and more studies are needed in order to better understand the mechanism of action of ORAS.

**Scheme 20:**
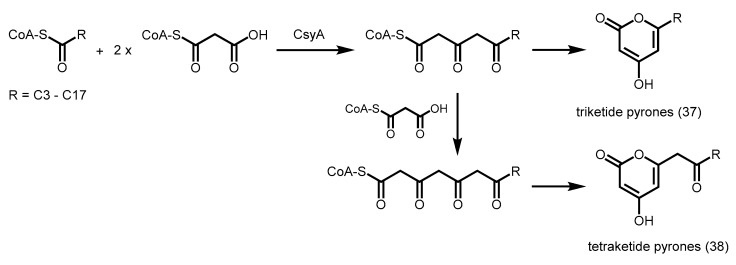
Synthesis of triketide and tetraketide pyrones catalyzed by *A. oryzae* CsyA.

Both *A. oryzae* CsyA and CsyB showed 38% identity with ORAS [85]. CsyA is likely to be a pyrone synthase (Scheme 20), capable of utilizing C4–C18 acyl-CoAs with malonyl-CoA as the extender to generate triketide pyrones. Furthermore, reactions with longer starters (C10–C18) resulted in the production of tetraketide pyrones.

**Scheme 21:**
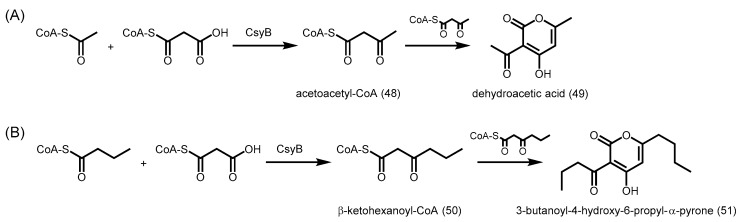
Synthesis of (**A**) dehydroacetic acid and (**B**) 3-butanoyl-4-hydroxy-6-propyl-α-pyrone catalyzed by *A. oryzae* CsyB.

CsyB is the first type III PKS capable of catalyzing the condensation of two diketide CoA esters, followed by cyclization to yield pyrones. CsyB was found to utilize acetoacetyl-CoA (**48**) both as a starter and as an extender to form dehydroacetic acid (**49**) (Scheme 21A) [86]. Similarly, two units of β-ketohexanoyl-CoA (**50**) can be coupled together by CsyB to form 3-butanoyl-4-hydroxy-6-propyl-α-pyrone (**51**) (Scheme 21B). Furthermore, CsyB is able to catalyze a single decarboxylative condensation of malonyl-CoA with C4–C10 acyl-CoAs to produce a β-keto fatty acyl intermediate, which can then undergo a second condensation with acetoacetyl-CoA to form 3-acetyl-4-hydroxy-6-alkyl-α-pyrones. If acetoacetyl-CoA is unavailable, CsyB is also able to catalyze the condensation of malonyl-CoA with acetyl-CoA to generate acetoacetyl-CoA (Scheme 21A). The broad substrate specificities and unique coupling reaction mechanisms of CsyB make it an ideal target for future structural studies.

**Figure 15:**
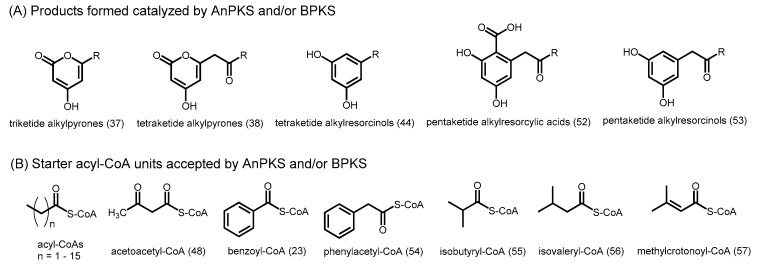
Substrate and product profiles of *A. niger* AnPKS and *B. cinerea* BPKS. (**A**) Products formed, and (**B**) starter acyl-CoAs accepted by the enzyme.

An unusual type III PKS with extremely broad substrate specificity was cloned from *Aspergillus niger*. *Aspergillus niger* polyketide synthase (AnPKS) shares 37% identity with ORAS, and homology modeling using the structure of ORAS as a reference reveals a similar acyl-binding tunnel [21]. Indeed, AnPKS was found to utilize a variety of acyl-CoAs (C2–C18) as starters and malonyl-CoA as the extender to generate triketide alkylpyrones. AnPKS was also able to form tetraketide alkylpyrones, tetraketide alkylresorcinols, and pentaketide alkylresorcinols **53** from C10–C18 acyl-CoAs (Figure 15A). Furthermore, acetoacetyl-CoA, the aromatic acyl-CoAs such as benzoyl-CoA and phenylacetyl-CoA (**54**), and branched acyl-CoAs such as isobutyryl-CoA (**55**), isovaleryl-CoA (**56**), and methylcrotonyl-CoA (**57**), are readily accepted by AnPKS (Figure 15B). The promiscuity of AnPKS makes it an ideal target for the engineered biosynthesis of unnatural polyketides with potential drug applications.

The *Botrytis cinerea* type III PKS (BPKS) was also recently found to accept short-chain to long-chain acyl-CoAs (C2–C18) as starters with malonyl-CoA as the extender to generate triketide alkylpyrones (Figure 15A) [87]. Tetraketide alkylpyrones were also formed from C10–C18 acyl-CoAs, while pentaketide alkylresorcylic acids **52** and pentaketide alkylresorcinols **53** were also produced from C16–C18 acyl-CoAs. Moreover, BPKS is able to generate a hexaketide alkylresorcylic acid from C18 acyl-CoA. BPKS is also able to utilize acetoacetyl-CoA and the aromatic benzoyl-CoA as starters to form triketide pyrones (Figure 15B). BPKS shares 65% identity with ORAS and similar to other type III PKSs which utilize long-chain acyl-CoAs as substrates, homology modeling studies show an acyl-binding tunnel in the active site. Recently, pyrones have been found to possess anti-cancer [88] and antibacterial [89] properties, making them potential lead structures for bioactive agents. However, more studies are needed to understand how differential chain extension and thus product profiles of BPKS and other fungal type III PKSs can be regulated based on the length of the starter units.

## 3. Precursor-Directed Biosynthesis of Polyketides

Although type III PKSs share a similar three-dimensional αβαβα fold and a conserved Cys-His-Asn catalytic triad, subtle differences in the active site cavities result in diverse substrate specificities and different product profiles. Notably, manipulation of the biosynthetic reaction by the incorporation of unnatural substrates has led to the generation of novel polyketide libraries. For instance when both the starters and the extenders were substituted with non-physiological substrates, CHS from the herb *Scutellaria baicalensis* was able to catalyze the formation of unnatural polyketides [90]. Other than the preferred substrates of *p*-coumaroyl-CoA and malonyl-CoA, *S. baicalensis* CHS was able to accept benzoyl-CoA as the starter and methylmalonyl-CoA as the extender to produce methylated triketide and tetraketide pyrones. Furthermore, the CHS also catalyzed the decarboxylative condensation of methylmalonyl-CoA with hexanoyl-CoA to yield a methylated triketide pyrone. CHSs from the Thai medicinal plant *Cassia alata* were also found to be promiscuous and accepted butyryl-CoA, isovaleryl-CoA, hexanoyl-CoA, benzoyl-CoA, cinnamoyl-CoA, and *p*-coumaroyl-CoA as starters to form triketide and tetraketide pyrones, and phloroglucinol derivatives in the presence of malonyl-CoA as the extender [91]. The ability of CHSs to utilize CoA esters that are not derived from the phenylpropanoid pathway highlights the role CHSs can play in synthesizing diverse bioactive polyketide compound libraries.

**Figure 16:**
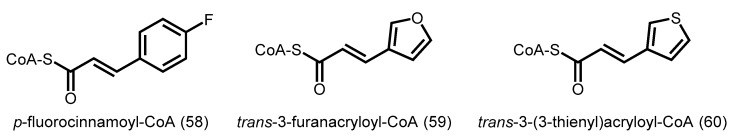
Starter acyl-CoA substrates utilized by *A. hypogaea* STS.

Similar to studies on CHSs, STS from *Arachis hypogaea* (peanut) was also found to accept several unnatural substrates to produce unique polyketides [92]. In particular, incubation of peanut STS with *p*-fluorocinnamoyl-CoA (**58**), *trans*-3-furanacryloyl-CoA (**59**), or *trans*-3-(3-thienyl)acryloyl-CoA (**60**, Figure 16) in the presence of malonyl-CoA yielded stilbene-like products.

When the peanut STS was incubated with other *p*-substituted cinnamoyl-CoA analogues (-Cl, -Br, and –OCH_3_), only triketide and tetraketide pyrones were obtained. This indicated potential steric effects by substituents that are larger than that of the wild-type substrate (-OH), which affected the stability and/or the conformation of polyketide intermediates in the cyclization pocket of the active site. Interestingly, when *p*-coumaroyl-CoA, cinnamoyl-CoA, or phenylpropionyl-CoA were utilized as starters, Scots pine STS was able to form pentaketide stilbenes in addition to tetraketide and triketide products, suggesting a considerable amount of flexibility in chain elongation in the active site cavity [93].

**Scheme 22:**
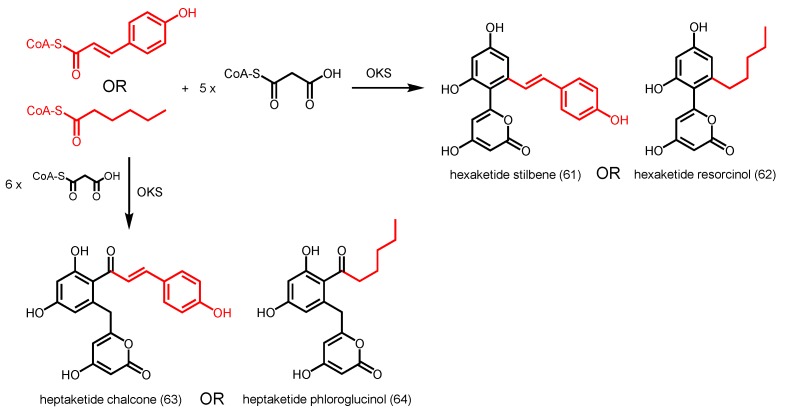
Synthesis of various polyketides catalyzed by *A. arborescens* OKS.

Octaketide synthase (OKS) from *A. arborescens* is known to catalyze seven sequential decarboxylative condensations of malonyl-CoA with an acetyl-CoA starter molecule to form the octaketides SEK4 (**12**) and SEK4b (**13**) (Scheme 2). Further studies on substrate tolerance revealed that aloe OKS is able to accept *p*-coumaroyl-CoA as a starter to generate a novel hexaketide stilbene (**61**) and heptaketide chalcone (**63**) in the presence of malonyl-CoA as the extender (Scheme 22) [94]. Similarly, OKS condenses malonyl-CoA with hexanoyl-CoA, followed by a C6 to C11 or C12 to C7 aldol-type cyclization to yield a hexaketide resorcinol (**62**) or heptaketide phloroglucinol (**64**) respectively (Scheme 22). The aloe OKS also utilizes long-chain C8–C20 acyl-CoAs as starters to form triketide and tetraketide α-pyrones [40]. This indicates that the ‘downward expanding’ active site architecture of aloe OKS is large enough to accommodate bulky substrates and intermediates. A similar octaketide-producing PKS (HpPKS2) from *Hypericum perforatum* (St. John’s wort) was also found to be promiscuous and accepted isobutyryl-CoA, hexanoyl-CoA, and benzoyl-CoA as starters to generate a series of triketide to heptaketide products [95].

**Scheme 23:**
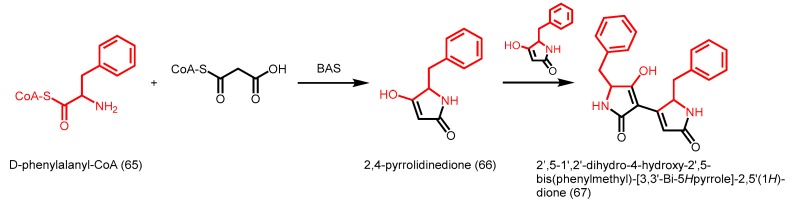
Novel polyketides catalyzed by *R. palmatum* BAS.

The rhubarb BAS catalyzes a single decarboxylative condensation of malonyl-CoA to *p*-coumaroyl-CoA, followed by another decarboxylation to form the diketide benzalacetone. As a result of a wide active site entrance caused by a F215L replacement (numbering in alfalfa CHS) (Figure 6B), rhubarb BAS is able to accept a unique range of substrates, including the bulky *N*-methylanthraniloyl-CoA as a starter with malonyl-CoA or methylmalonyl-CoA as the extender to yield quinolone alkaloids [96,97]. Furthermore, the promiscuous enzyme is able to catalyze the decarboxylative condensation of malonyl-CoA with phenylalanyl-CoA (**65**), followed by intramolecular lactamization to form 2,4-pyrrolidinedione (**66**). 2,4-pyrrolidinedione and its analogs may undergo further spontaneous dimerization (Scheme 23) [97]. Interestingly, the tetramic acid dimer, 2′,5-1′,2′-dihydro-4-hydroxy-2′,5-bis(phenylmethyl)-[3,3′-Bi-5Hpyrrole]-2,5′(1*H*)-dione (**67**), exhibited moderate cytotoxic activity against murine leukemia P388 cells, thus highlighting the utility of precursor-directed biosynthesis.

Numerous studies have illustrated the feasibility of performing precursor-directed biosynthesis using type III PKSs to generate novel polyketides. Indeed, by establishing a combinatorial biosynthetic route in *Escherichia coli* [98] and exploring the substrate promiscuity of a mutant CHS with STS activity (18xCHS) from alfalfa [26], at least 413 potentially novel polyketide compounds were biosynthesized (manuscript in preparation). In this approach, various combinations of 69 starter acyl-CoA and 12 extender acyl-CoA precursors generated by several promiscuous acid-CoA ligases were delivered to the mutant CHS, and the substrate profile was established. Similar work in precursor-directed biosynthesis using a chalcone synthase from *O. sativa* led to the development of novel polyketides as antimicrobial drug leads [28]. The structural and chemical diversity of products make type III PKSs an excellent platform for the engineered biosynthesis of novel bioactive polyketides. More structural variants of starter and extender substrates can be tested in order to generate comprehensive libraries of compounds which may serve as potential drug leads.

## 4. Insights into Mutagenesis Studies

Crystallographic analyses of the plant type III PKSs (alfalfa CHS [23], Scots pine STS [26], daisy 2-PS [35], rhubarb BAS [45], *O. sativa* CUS [49], *C. microcarpa* ACS and QNS [59], and brown alga phloroglucinol synthase [60]), the bacterial PKSs (*S. coelicolor* THNS [66], *M. tuberculosis* PKS11 [73] and PKS18 [72], *A. vinelandii* ArsC [80]), and of the fungal ORAS from *N. crassa* [83] have enabled the understanding of the basis of starter molecule selectivity, chain elongation, and cyclization of the linear polyketide intermediate. These structural studies have provided insights into the mechanistic details of type III PKSs, illustrating how subtle modifications in the active site cavity can expand the biosynthetic repertoire and result in the generation of diverse products. Manipulation of active site residues by random mutagenesis [68] and/or site-directed mutagenesis has proved useful in the understanding of and subsequently, engineering of the substrate specificity and activity of type III PKSs.

### 4.1. Dissection of Catalytic and Structural Roles of Residues via Mutagenesis

**Figure 17:**
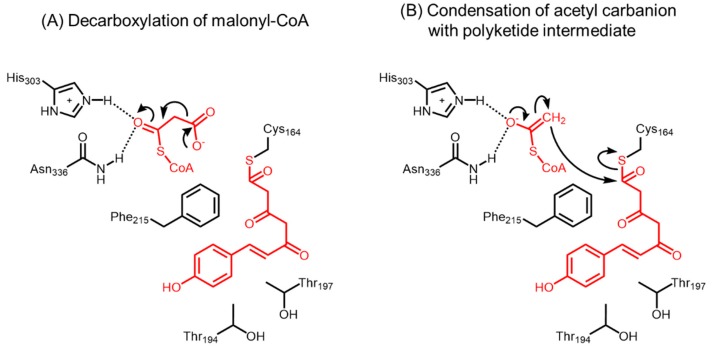
(**A**) Decarboxylation of the extender malonyl-CoA is catalyzed by His303 and Asn336 in alfalfa CHS. Phe215 is thought to orient the terminal carboxylate of malonyl-CoA and aid in the reaction; (**B**) The acetyl carbanion subsequently undergoes a condensation with the enzyme-bound triketide intermediate.

The active site of alfalfa CHS (PDB ID: 1CGK) was found to be situated at the intersection of a CoA-binding tunnel and a bi-lobed cavity, which comprises a coumaroyl-binding pocket and a cyclization pocket [27]. Site-directed mutagenesis of the catalytic Cys164 to alanine, serine, or aspartate resulted in no naringenin production, confirming the role of Cys164 as the nucleophile and attachment site for the starter unit [25]. Mutations of Cys164 only modestly affected the malonyl-CoA decarboxylation activity (C164A and C164S), whereas mutations of Phe215, His303, and Asn336 abolished (H303A, H303D, H303N, H303T, N336H, and N336Q) or severely impaired (F215S, F215W, F215Y, N336A, N336D, and N3336K) malonyl-CoA decarboxylation activities, except for the isosteric mutation of His303 to a glutamine. Hence, Cys164 is not important for malonyl-CoA decarboxylation and replacing the nucleophile with a neutral alanine residue only prevents the loading of the starter molecule. The ability of His303 to hydrogen bond with the thioester carbonyl oxygen of the extender substrate and stabilize the negative charge in the acetyl carbanion was found to be essential for malonyl-CoA decarboxylation (Figure 17). In addition, Cys164, Phe215, and His303 mutants did not significantly affect the K_d_ values of CoA or acetyl-CoA, suggesting that these residues do not contribute to binding of CoA esters in the active site. Although the binding of CoA was not considerably affected, all Asn336 mutants except N336H showed a 15–40 fold increase in K_d_ values for acetyl-CoA binding compared to wild-type CHS, thus indicating that this residue contributes to the binding of substrates with a thioester carbonyl. The hydrogen bond between Asn336 and the thioester carbonyl oxygen of malonyl-CoA serves as a guide to ensure the proper conformation for interaction with His303, and for decarboxylation and subsequent condensation of the acetyl carbanion to the enzyme-bound starter unit or polyketide intermediate (Figure 17). Similarly, Phe215 may orient the terminal carboxylate of the extender via van der Waals forces, and aid in malonyl-CoA decarboxylation and chain extension of the polyketide intermediate.

Mutagenesis studies have also identified various non-catalytic residues lining the active site cavity that are believed to be important in determining the chain length specificity and the substrate and product profiles of PKSs. These residues include Thr132, Ser133, Thr194, Thr197, Gly256, Phe265, and Ser338 (numbering in alfalfa CHS) which are typically replaced in other type III PKSs but are conserved in CHSs [27]. Daisy 2-pyrone synthase (2-PS) shares the same overall structure and catalytic triad as alfalfa CHS, but variations in three residues (T197L, G256L, and S338I) lining the active site cavity results in a significant reduction in the active site volume [35]. The 2-PS structure was the first to demonstrate a simple means of functional divergence in type III PKSs, whereby steric restriction could alter the selectivity of the starter molecule, the number of chain elongation reactions, and the cyclization chemistry employed to terminate and release the polyketide product.

In *H*. *androsaemum* benzophenone synthase (BPS), a T132L mutation (numbering in alfalfa CHS) resulted in changes in the active site cavity which limited polyketide chain extension and transformed the enzyme from a biphenyl synthase into a phenylpyrone synthase with a trace of the wild-type activity [99]. In another study, structure-based mutagenic transformation of alfalfa CHS resulted in a change in chain termination chemistry from a Claisen condensation typical of CHSs to an aldol condensation usually catalyzed by STSs, indicating that functional divergence can be determined by electronic factors rather than steric effects of the active site cavity [26].

Aloe contains two highly similar type III PKSs, pentaketide chromone synthase (PCS) and octaketide synthase (OKS), which share more than 90% identity with each other. Although these two enzymes possess different biochemical functions, a single mutational change of Met197 in the pentaketide-producing PCS to Gly197 in OKS (numbering in alfalfa CHS) resulted in the synthesis of the octaketides, SEK4 (**12**) and SEK4b (**13**) (Scheme 2) [38]. Conversely, a reciprocal mutation in aloe OKS to the bulky methionine residue resulted in a loss of octaketide synthase activity and a gain in pentaketide synthase activity [40]. In addition, various small-to-large substitutions in OKS (G197A, G197T, G197M, G197L, G197F, and G197W) resulted in a loss of octaketide production and a concomitant generation of shorter chain length polyketides (from heptaketide to triketide), depending on the steric bulk of the side chain. Therefore, residue 197 is likely to control polyketide chain length and product specificity. The reduced steric bulk due to the M197G substitution in the PCS mutant unlocks two buried pockets, thus expanding the available cyclization cavity to accommodate the octaketide product [38]. Interestingly, the location and orientation of one of the pockets of the PCS M197G mutant superimpose well with a similar acyl-binding tunnel in OKS [94], in bacterial type III PKSs such as THNS [66] and PKS18 [72], and in the fungal 2′-oxoalkylresorcylic acid synthase (ORAS) [83]. Since aloe OKS can utilize long-chain (C6–C20) acyl-CoAs to form triketide and tetraketide pyrones [40], it is possible that the plant, bacterial, and fungal type III PKSs share a similar active site architectural strategy to synthesize the long-chain polyketides such as pyrones, phloroglucinols, and resorcinols.

### 4.2. Structure-Based Engineering and the Versatility of Type III PKSs

Manipulation of the type III PKS reactions by using rationally engineered mutant enzymes in conjunction with substrate analogues can result in a further generation of chemically and structurally distinct unnatural polyketides. The ‘gatekeeper’ Phe215 is generally conserved in most type III PKSs and is located between the CoA-binding tunnel and the entrance of the active site cavity. Despite having a lower malonyl-CoA decarboxylation activity compared to wild-type alfalfa CHS, the F215S mutant is able to utilize *p*-coumaroyl-CoA, phenylacetyl-CoA, benzoyl-CoA, and C5–C8 acyl-CoAs with less than 3% the catalytic efficiency of the wild-type enzyme to produce tetraketide lactones [100]. Intriguingly, although *N*-methylanthraniloyl-CoA is not normally utilized by the wild type CHS, the F215S mutant was found to accept the bulky starter with a comparable *k*_cat_/K_m_ to *R. graveolens* acridone synthase (ACS), to form a tetraketide *N*-methylanthraniloyltriacetic acid lactone (**31**). A modification into a serine residue at position 215 results in a reduction in steric hindrance and a wider active site entrance, thus allowing the entry of the bulky *N*-methylanthraniloyl-CoA.

Although octaketides are not usually synthesized by the wild type *S. baicalensis* CHS, the S338V mutant CHS was found to produce trace amounts of SEK4 (**12**) and SEK4b (**13**) using eight units of malonyl-CoA (Scheme 2C) [101]. This S338V mutation was proposed to facilitate the extension of the linear polyketide intermediate into a buried pocket in the active site cavity of the enzyme by providing steric guidance. It was demonstrated for the first time that CHS could be engineered to generate longer polyketides such as octaketides. Furthermore, the synthesis of SEK4 (**12**) and SEK4b (**13**) by *S. baicalensis* CHS was dramatically improved in an OKS-like triple mutant (T197G/G256L/S338V). These results suggested that rational site-directed mutagenesis efforts can be used to generate CHSs with different substrate selectivities and altered product profiles, thereby producing diverse novel polyketides.

**Scheme 24:**
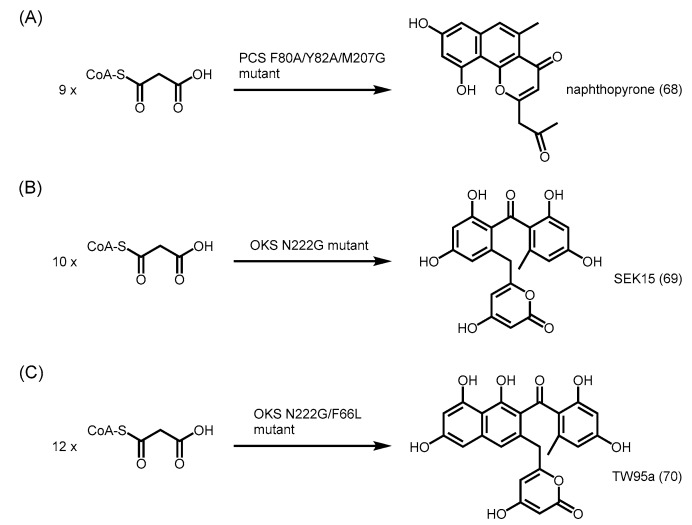
Novel polyketides biosynthesized by mutant type III PKSs. (**A**) PCS F80A/Y82A/M207G mutant catalyzes the formation of napthopyrone; (**B**) OKS N222G mutant catalyzes the formation of SEK15; and (**C**) OKS N222G/F66L mutant catalyzes the formation of TW95a.

Structure-based engineering efforts on the aloe PCS and OKS further extended the biosynthetic repertoire of type III PKSs. When two aromatic residues at the bottom of the buried pocket of the PCS M207G mutant (corresponding to Thr197 in alfalfa CHS) were simultaneously replaced by alanine, the active site cavity was further expanded by approximately four times compared to the wild-type PCS [102]. Interestingly, the PCS F80A/Y82A/M207G mutant was able to catalyze the decarboxylative condensation of nine units of malonyl-CoA with an altered mechanism of cyclization to yield an unnatural nonaketide naphthopyrone (**68**) (Scheme 24A). Similarly in OKS, when Asn222 situated at the bottom of the elongation cavity was replaced by a glycine residue, a novel decaketide benzophenone SEK15 (**69**) was produced from ten units of malonyl-CoA (Scheme 24B) [94]. The N222G mutant OKS was also able to accept phenylacetyl-CoA or benzoyl-CoA as starters to form novel octaketides and heptaketide benzophenone respectively [103]. Simultaneous mutation of the neighbouring Phe66 residue to leucine resulted in an OKS double mutant that is capable of synthesizing an unnatural dodecaketide naphthophenone TW95a (**70**) by the successive decarboxylative condensations of 12 units of malonyl-CoA, making it one of the longest polyketide synthesized by the structurally simple type III PKS (Scheme 24C) [104].

**Scheme 25:**
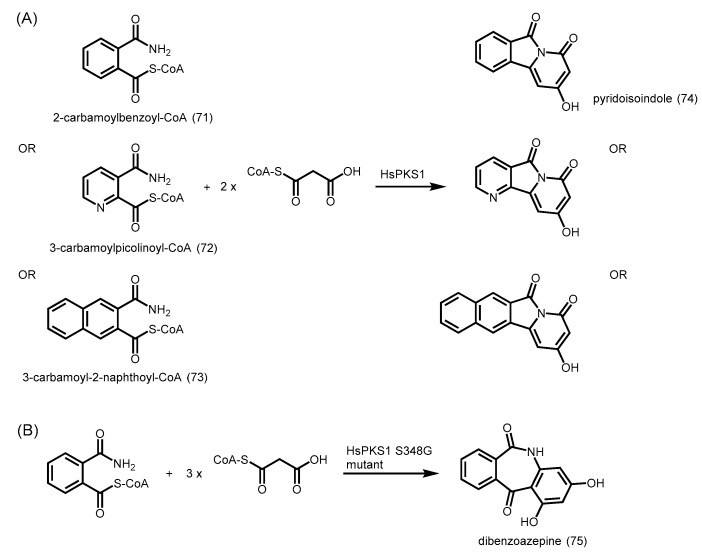
Synthesis of polyketide-alkaloid hybrids catalyzed by (**A**) *H. serrata* HsPKS1 and its (**B**) S348G mutant.

A recently discovered CHS from a primitive club moss *Huperzia serrata* (HsPKS1) was found to display unusually broad substrate selectivity and produced unnatural polyketide-alkaloid hybrid molecules [105]. Using precursor-directed approaches, HsPKS1 synthesized a novel pyridoisoindole (**74**) from the condensation of 2-carbamoylbenzoyl-CoA (**71**), a synthetic nitrogen-containing non-physiological starter, with two units of malonyl-CoA (Scheme 25A). Conversely, the promiscuous aloe OKS was only able to generate a tetraketide pyrone as a minor product from the bulky synthetic starter. HsPKS1 was also able to generate novel alkaloids from the starters, 3-carbamoylpicolinoyl-CoA (**72**) and 3-carbamoyl-2-naphthoyl-CoA (**73**) (Scheme 25A). The structure-based S348G mutant (corresponding to Ser338 in alfalfa CHS) results in an expansion of the active site cavity which not only extended the polyketide chain length, but also altered the cyclization mechanism to yield a biologically active, ring-expanded dibenzoazepine (**75**) from the condensation of 2-carbamoylbenzoyl-CoA with three units of malonyl-CoA (Scheme 25B).

Remarkably, the dibenzoazepine alkaloid inhibited biofilm formation by methicillin-susceptible *Staphylococcus aureus* (MIC = 12.0 μg/mL). Hence, structure-based engineering of type III PKSs coupled with precursor-directed biosynthesis using rationally designed substrate analogues can provide an excellent platform for the further development of unnatural novel polyketides with unique therapeutic functions.

Studies on *S. griseus* RppA showed that the bulky Tyr224 (corresponding to Gly256 in alfalfa CHS) is important for RppA to select malonyl-CoA as a starter unit [106]. Mutations at this site (Y224A, Y224C, Y224G, and Y224L) led to a decrease in competition for malonyl-CoA and the mutant enzyme was able to utilize phenylacetyl-CoA or hexanoyl-CoA instead to synthesize tetraketide and triketide pyrones. This is analogous to the G256L replacement in rhubarb ALS which utilizes acetyl-CoA as a starter instead of the bulky *p*-coumaroyl-CoA, suggesting that the residue at position 256 controls starter unit selectivity [37]. Similar Tyr224 mutations in *S. coelicolor* THNS showed that the mutant enzyme can produce triketide pyrones from acetyl-CoA, acetoacetyl-CoA, hexanoyl-CoA, and benzoyl-CoA starters in significantly higher amounts compared to the wild-type THNS, due to a decrease in loading efficiency of the malonyl-CoA starter [107]. In addition, Ala305 (corresponding to Ser338 in alfalfa CHS) was found to be important for chain elongation, as a bulky 2-PS-like A305I mutation in *S. griseus* RppA resulted in the generation of triketide pyrones only from phenylacetyl-CoA or hexanoyl-CoA starters [106]. These results indicated that the overall backbone architecture of the active site cavity of RppA is similar to that of plant type III PKSs, thus structure-based engineering efforts in plant PKSs are likely to translate well to bacterial type III PKSs with minor differences.

## 5. Conclusions

Recently identified type III PKSs that possess novel catalytic activities, such as quinolone synthase (QNS), *Rhodospirillum* polyketide synthase (RpsA), and *Botrytis cinerea* type III PKS (BPKS), can provide unique polyketide scaffolds for synthetic enzymology. Until recently, biosynthesis of novel polyketide libraries has been dependent upon the broad substrate specificities of the type III PKSs. By rationally incorporating amino acid substitutions, the active site cavity of type III PKSs can be altered or expanded to facilitate the synthesis of more complex polyketides. This characteristic makes type III PKSs great candidates for structure-based engineering approaches to expand the chemical and structural diversity of natural products derived from these systems. Further studies on the catalytic potential and versatility of the functionally divergent type III PKSs should provide more intimate structural details of the biosynthetic processes.

Since the diversity of the polyketides biosynthesized by PKSs is partly dependent on the starter and extender units, variations in the choice of starters and extenders is important. However, there has been a lack of a toolkit that describes the means to introduce novel acyl-CoA precursors to PKSs for polyketide biosynthesis. Instead of chemical synthesis, an alternative way to generate a wide range of novel acyl-CoA esters is to make use of promiscuous acid-CoA ligases with unique carboxylic acid scaffolds [98]. By determining substrate promiscuities of acid-CoA ligases and PKSs beyond conventional substrate pools, we can establish novel enzymatic routes for the biosynthesis of unnatural polyketides which can be subsequently applied to drug screening efforts. These studies serve as a primer towards the development of novel and valuable polyketides with therapeutic potential via synthetic enzymology.

## Abbreviations

The following abbreviations are used in this manuscript:

ACP	Acyl carrier protein
ACS	Acridone synthase
ALS	Aloesone synthase
AnPKS	*Aspergillus niger* polyketide synthase
ARAS2	Alkyl-resorcylic acid synthase 2
BAS	Benzalacetone synthase
BBS	Bibenzyl synthase
BIS	Biphenyl synthase
BPS	Benzophenone synthase
CHS	Chalcone synthase
CoA	Coenzyme A
CsyA	Chalcone synthase-like A
CTAL	Coumaroyl triacetic acid lactone
CTAS	*p*-coumaroyl triacetic acid synthase
CURS	Curcumin synthase
CUS	Curcuminoid synthase
DCS	Diketide-CoA synthase
DpgA	3,5-dihydroxyphenylacetic acid synthase
GCS	Germicidin synthase
NAC	*N*-acetylcysteamine
OKS	Octaketide synthase
ORAS	2′-oxoalkylresorcylic acid synthase
PCS	Pentaketide chromone synthase
PhlD	Phloroglucinol synthase
PKS	Polyketide synthase
PpASCL	*Physcomitrella patens* anther-specific chalcone synthase-like enzyme
2-PS	2-pyrone synthase
QNS	Quinolone synthase
STCS	Stilbenecarboxylate synthase
STS	Stilbene synthase
THNS	1,3,6,8-tetrahydroxynaphthalene synthase
